# Island plants with newly discovered reproductive traits have higher capacity for uniparental reproduction, supporting Baker’s law

**DOI:** 10.1038/s41598-024-62065-4

**Published:** 2024-05-18

**Authors:** Barbara Keller, Barbara Alther, Ares Jiménez, Konstantina Koutroumpa, Emiliano Mora-Carrera, Elena Conti

**Affiliations:** 1 Department of Systematic and Evolutionary Botany, University of Zurich, Zurich, Switzerland; 2 Botanischer Garten und Botanisches Museum Berlin (BGBM), Freie Universität Berlin, Berlin, Germany

**Keywords:** Plant ecology, Plant evolution, Plant reproduction

## Abstract

Uniparental reproduction is advantageous when lack of mates limits outcrossing opportunities in plants. Baker’s law predicts an enrichment of uniparental reproduction in habitats colonized via long-distance dispersal, such as volcanic islands. To test it, we analyzed reproductive traits at multiple hierarchical levels and compared seed-set after selfing and crossing experiments in both island and mainland populations of *Limonium lobatum*, a widespread species that Baker assumed to be self-incompatible because it had been described as pollen-stigma dimorphic, i.e., characterized by floral morphs differing in pollen-surface morphology and stigma-papillae shape that are typically self-incompatible. We discovered new types and combinations of pollen and stigma traits hitherto unknown in the literature on pollen-stigma dimorphism and a lack of correspondence between such combinations and pollen compatibility. Contrary to previous reports, we conclude that *Limonium lobatum* comprises both self-compatible and self-incompatible plants characterized by both known and previously undescribed combinations of reproductive traits. Most importantly, plants with novel combinations are overrepresented on islands, selfed seed-set is higher in islands than the mainland, and insular plants with novel pollen-stigma trait-combinations disproportionally contribute to uniparental reproduction on islands. Our results thus support Baker’s law, connecting research on reproductive and island biology.

## Introduction

Flowering plants display great diversity of reproductive systems involving either two parents, through which outcrossed progeny is formed, or only one parent, through which selfed and clonal progeny is formed. In species with hermaphroditic flowers capable of producing both outcrossed and selfed progeny, outcrossing frequencies can range from zero to one^1^. Sustained high levels of uniparental reproduction reduce genetic variation in populations, limiting their ability to adapt to environmental changes^2,3^. Moreover, selfed progeny is frequently less fit than outcrossed progeny (i.e., inbreeding depression) due to increased homozygosity of alleles at loci with recessive deleterious mutations and/or heterozygote advantage^4,5^. Therefore, a wide range of floral traits have evolved to promote outcrossing, including different types of floral heteromorphy^6,7^.

Conversely, uniparental reproduction provides reproductive assurance when mates and/or pollinators are absent or scarce^8–10^. Uniparental reproduction can be advantageous when plants colonize distant, isolated habitats, including volcanic islands formed de novo through tectonic processes, via long distance-dispersal across oceanic barriers^11,12^. The notion that individuals capable of uniparental reproduction are more successful long-distance colonizers than individuals dependent on biparental reproduction has fascinated evolutionary biologists since it was first postulated by Baker^13–16^ for plants and Longhurst^17^ for animals, an idea later termed “Baker’s law” by Stebbins^9^. While Baker and Longhurst provided verbal models, Pannell et al.^12^ generated testable hypotheses emphasizing the role of mate rather than pollinator limitation, especially for long-distance colonization of oceanic islands by plants^18,19^. They also underscored that adaptive evolution after colonization might change the original traits favoring long-distance colonization and initial establishment, implying that intra-specific studies of species occurring on both islands and mainland may be more suitable to test the predictions of Baker’s law than inter-specific comparisons. Summarizing, Baker’s law, as specified by Pannell et al.^12^ provides the general framework to test whether the ability for uniparental reproduction is indeed overrepresented in habitats colonized via long-distance dispersal, such as oceanic islands.

Uniparental reproduction is common in plants and occurs either sexually via self-fertilization (i.e., autogamy) or asexually via vegetative propagation or apomixis, the latter term referring to clonal reproduction through seeds^20^. Plants can reproduce via self-fertilization if they are self-compatible (SC), that is, if pollen can germinate and fertilize the ovules of the same plant (i.e., facultative selfers). Conversely, fully self-incompatible (SI) plants, i.e., plants where pollen of the same plant is rejected, can only reproduce via outcrossing (i.e., obligate outcrossers)^1^. However, plant species are not always uniformly SC or SI. Indeed, some species exhibit intra-specific variation in the strength and frequency of SI, either due to plastic environmental responses of SI or the spread of mutations that weaken or disable SI genes^21–26^.

If Baker’s law applies, then plants that can propagate uniparentally via selfing, apomixis, or vegetative propagation should be more capable of colonization after long-distance dispersal, hence should be overrepresented in isolated habitats. Therefore, species with populations in both mainland and volcanic islands are particularly well suited to test whether successful establishment on islands after long-distance dispersal is associated with increased capability for uniparental reproduction, regardless of whether it is aided by pollinators or not (i.e., autonomous selfing). The increase of uniparental reproduction predicted by Baker’s law can be detected as higher production of selfed seed enabled by higher levels of SC in island than mainland plants. To our knowledge, controlled crossing experiments aimed at comparing levels of uniparental reproduction in island vs. mainland populations of the same species have been performed in only three cases, all from the New World: *Lycium carolinianum* (*Solanaceae*: Hawaiian Islands and North America)^18^, *Waltheria ovata* (*Malvaceae*: Galapagos Islands and Ecuador)^27^, and the invasive *Oeceoclades maculata* (*Orchidaceae*: Puerto Rico and Brazil)^28^. The intra-specific studies mentioned above found that the capacity for uniparental reproduction is enriched on islands, supporting Baker’s law.

Baker developed the ideas behind his namesake law by studying the *Limonieae* tribe (comprising 22 genera, among which *Limonium, Armeria*, and *Myriolimon*, mentioned below) of *Plumbaginaceae*^29^, where he described a new type of floral dimorphism (see below) and performed self- and cross-pollination experiments aimed at determining whether plants were self-incompatible or self-compatible^13–16^. *Limonieae* include three different reproductive strategies that can vary both intra- and interspecifically: sexual reproduction with SI, sexual reproduction with SC, and apomixis, the last often associated with polyploidy in the tribe^6,14–16,30^.

**Figure 1:**
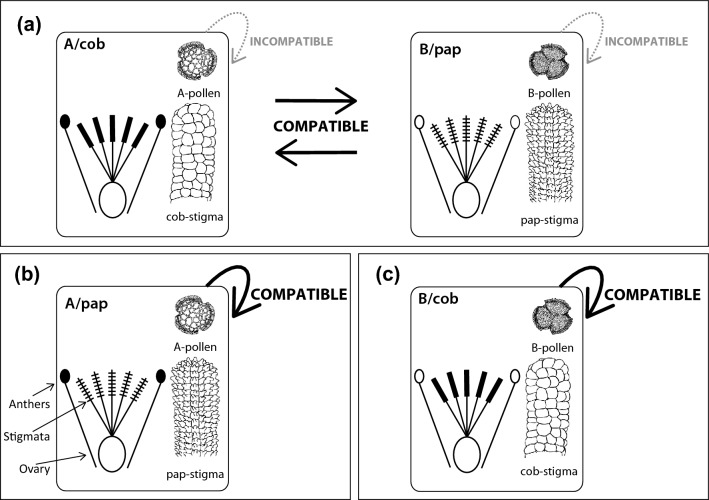
Schematic drawings of previously described combinations of male and female organs in sexually reproducing *Limonieae*: (**a**) dimorphic species comprising self- and intra-morph incompatible (but inter-morph compatible) A/cob (with A pollen and cob stigmas) and B/pap (with B pollen and pap stigmas) flowers on separate individuals; (**b**) monomorphic species comprising self- and intra-morph compatible A/pap (with A pollen and pap stigmas) flowers; and (**c**) monomorphic species comprising self- and intra-morph compatible B/cob (with B pollen and cob stigmas) flowers. Filled and empty anthers represent A and B pollen, respectively. Solid cross lines on stigmas indicate protruding, or papillate (pap) papillae, absence of cross lines indicates flat (cob) papillae. Drawings of pollen grains and stigmas were modified from Baker^16^. Baker considered *L. lobatum* as dimorphic and self-incompatible but did not provide any details on the number and provenance of the plants he studied^14,15^. Therefore, only plants with A/cob and B/pap flowers were previously known from this species.

Typical SI sexual reproduction is associated with a pollen-stigma dimorphism characterized by two genetically determined floral morphs that are self- and intra-morph incompatible and occur at roughly equal frequencies in populations (i.e., isoplethy). Plants of the so-called A/cob floral morph have pollen with coarse exine sculpturing (A pollen) and stigmas with flat (i.e., cob) papillae. Plants of the so-called B/pap floral morph have pollen with fine exine sculpturing (B pollen) and stigmas with protruding (i.e., papillate = pap) papillae (Fig. 1a)^14,16,31^. In pollen-stigma dimorphic species, topological complementarity between pollen and stigma morphologies might promote adherence of compatible *vs*. rejection of incompatible pollen grains^32,33^. Consequently, the combination of pollen-stigma dimorphism with SI, which is thought to be sporophytically controlled, prevents pollen from adhering and germinating on stigmas of both the same individual and the same floral morph, favoring inter-morph mating, hence outcrossing^6,32,33^.

Conversely, typical pollen-stigma monomorphism is associated with self-compatibility (SC sexual reproduction): some taxa consist entirely of A/pap plants with A pollen and pap stigmas (Fig. 1b), while other taxa consist entirely of B/cob plants with B pollen and cob stigmas (Fig. 1c)^6,32,33^. The mating system of monomorphic, SC *Limonieae* has been investigated only in *Limonium carolinianum*, shown to be a mixed-mating species^34^.

Finally, apomictic *Limonieae* produce either misshaped and irregularly sized A or B pollen grains of reduced viability or, rarely, no pollen^14,35^. Apomicts reproduce via unfertilized seeds (obligate agamospermy)^6^, or, occasionally, also sexually (facultative agamospermy). Thus, apomixis can occur in populations monomorphic for any pollen-stigma combination (usually A/cob or B/pap and rarely B/cob or A/pap) and/or dimorphic populations (A/cob and B/pap)^6,14,36–38^. To summarize, previous studies revealed a close relationship between pollen-stigma combinations and reproductive strategies^14,32,33^. Therefore, combinations of pollen and stigma traits have been widely used to infer whether plants are SI sexual, SC sexual, or apomictic.

Concerning the genetics of pollen-stigma dimorphism (with associated SI), previous crossing experiments imply that, similarly to species with other types of floral dimorphism, it is sporophytically controlled by a few, tightly linked genes probably forming a supergene^6,32,33^. The most recent common ancestor of the tribe *Limonieae* was most likely sexual, SI, and pollen-stigma dimorphic^31^. Consequently, SI can be weakened or lost via mutations and/or recombination in the genomic regions controlling the floral dimorphism^39,40^, likely resulting in A/cob and B/pap plants with a weakened/disabled SI system or B/cob and A/pap plants with compatible pollen-stigma combinations, respectively. However, the genes controlling the pollen-stigma dimorphism and mutations responsible for its loss remain unknown.

Species displaying intra-specific diversity of reproductive traits and occurring on both the mainland and volcanic islands are especially suitable to test Baker’s law, because less divergence that could change the reproductive traits facilitating initial long-distance colonization is expected to occur within than between species. In *Limonieae*, intraspecific diversity of reproductive traits and SI has been described, to our knowledge, only in *Armeria maritima* and *Myriolimon ferulaceum*^30,37,41,42^. Specifically, several SI and SC subspecies or varieties have been reported in the highly variable species complex of *A. maritima*. Interestingly, in a few populations of the dimorphic *A. maritima ssp*. *maritima*, some individuals with pollen-stigma combinations traditionally regarded as SI (i.e., A/cob and B/pap) set seeds autonomously (i.e., self-fertilization unaided by pollinators), implying that they have a weakened/disabled SI system^6,30,43–45^. Additionally, few populations monomorphic for B/cob have been reported in the usually dimorphic *Myriolimon ferulaceum*^46^. However, no study has yet quantified the diversity of pollen and stigma traits in both island and mainland populations of species characterized by the pollen-stigma trait-combinations described above or experimentally tested whether the capacity for uniparental reproduction in such species may be overrepresented on islands, as predicted by Baker’s law. This gap of knowledge is investigated here using *Limonium lobatum* as model system because such species was central to the development of Baker’s law (see below).

*Limonium lobatum* (L. fil.) Chaz. (synonym *L. thouinii* (Viv.) Kuntze; *L.* sect*. Pteroclados* subsect. *Odontolepidea*; Tribe *Limonieae*; *Plumbaginaceae*) is a diploid (*2n* = 12), annual herb up to 50 cm tall with conspicuously winged, photosynthetic flowering stems bearing few dozens to hundreds of pentamerous flowers with pale-yellow corollas and pale-blue to whitish papery calyces that are visited by a set of generalist insects including bees and butterflies (Supplementary Fig. S1)^29,37,46–48^. The female reproductive structures (i.e., gynoecium) consist of one ovary with a single ovule and five styles (ca. 3 mm long) each subtending an elongated, filiform stigma (ca. 2 mm long; Supplementary Fig. S2a). The male reproductive structures (i.e., androecium) comprise five anthers fused to the base of the corolla but free at the top^37,47^. The architecture of the inflorescence is complex. Each inflorescence comprises several spikes, each spike comprises 6–12 spikelets, and each spikelet bears 2–3 flowers (Supplementary Fig. S2b). Within spikes and spikelets, flowers bloom from the proximal to the distal part. The corolla, stigmas, and anthers wither within less than 24 h after flower opening, while the papery calyx persists throughout the growing season. *Limonium lobatum* typically grows on sandy or gravelly soils in dry areas along the coastlines of the Mediterranean Basin (Algeria, Egypt, Greece, Iran, Iraq, Kuwait, Lebanon-Syria, Morocco, Palestine, Saudi Arabia, Sinai, Spain, Tunisia, Western Sahara, potentially also Aegina Island, and Kerkennah Island), extending also to Fuerteventura and Tenerife, two islands of the Canarian archipelago at the western end of its distributional range^14,15,47,49–52^. Island and mainland plants usually occur in small patches with a maximum radius of 100 m in populations of about 100–200 individuals. No dated phylogenies of *Limonium* in relation to its biogeographic history have been inferred to determine when and from where *L. lobatum* colonized the Canary Islands.

*Limonium lobatum* was regarded as a pollen-stigma dimorphic species, hence assumed to be SI, by Baker^14,15^. However, Baker did not provide any details on the number and provenance of the plants he examined. He was puzzled by the fact that this species (known under a different name at his time) with a large disjunction between mainland and Canary Islands was described as dimorphic, hence presumed SI: “*L. thouinii (Viv.) Kuntze is of particular interest because of its distribution from the Middle East throughout the Mediterranean lands (particularly on the North African side) to the Canary Isles. Despite the widespread distribution, including the large Atlantic disjunction, the species has kept its dimorphism of pollen and stigmata*” p. 616^15^. To paraphrase, Baker wondered how a species known as pollen-stigma dimorphic, hence presumed SI, could have colonized the Canary Islands via long-distance dispersal. However, recent field surveys of Canarian and Iberian plants of *L. lobatum* suggested that the species might harbor a diversity of pollen and stigma traits never described before (A. Jiménez, pers. obs.), hence may not be typically SI. Therefore, *L. lobatum* warrants in-depth analyses of reproductive traits and production of selfed vs. outcrossed seeds between island and mainland populations to test whether the ability for uniparental reproduction is enriched on islands, as predicted by Baker’s law.

**Figure 2:**
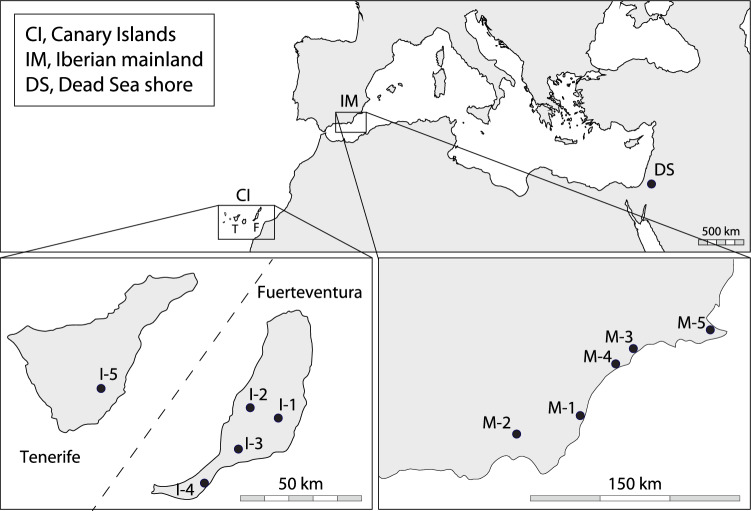
Locations of the eleven populations of *Limonium lobatum* sampled for this study. Locations of the five populations from the Canary Islands (Antigua: I-1; Betancuria: I-2; Tarajalejo: I-3; Esquinzo: I-4; and El Viso: I-5), the five populations from the Iberian mainland (Castillo de Macenas: M-1; Tabernas: M-2; Cala Blanca: M-3; Los Cocedores: M-4; and Los Nietos: M-5), and the single population from the Dead Sea shore. From the Canary Islands and the Iberian mainland, 20 experimental plants were raised from each population, while from the Dead Sea population only ten experimental plants could be raised (see Supplementary Fig. S5). Maps modified from https://d-maps.com/.

Using plants raised under standard greenhouse conditions from seeds randomly collected in natural island and mainland populations of *L. lobatum* (Fig. 2 and Supplementary Fig. S3; Supplementary Table S1), we analyzed pollen and stigma traits at different hierarchical levels (from intra-flower to inter-population) and tested Baker’s law by comparing island and mainland populations for seed-set after manual self- and cross-pollinations. Since initial colonizers lack mates after long-distance dispersal to volcanic islands, plants that can produce seeds uniparentally, whether with the aid of pollinators or not, should be overrepresented on islands vs. mainland. Specifically, we tested whether: (i) *L. lobatum* harbors undescribed diversity of pollen-stigma combinations; (ii) newly discovered combinations of reproductive traits, if found, are overrepresented on islands; and (iii) island plants have enhanced capacity for uniparental reproduction compared to mainland plants. Contrary to previous reports by Baker^14,15^, we discovered that *L. lobatum* is not a typical pollen-stigma dimorphic, SI species. Rather, it encompasses newly discovered types of pollen-stigma combinations, especially in island plants, which produce more selfed seed than mainland plants, conformant with the predictions of Baker’s law. The results of our experiments support the conclusion that *L. lobatum* is largely SC, rather than fully SI, as previously reported by Baker, and that the occurrence of self-compatibility might have enabled this species to colonize the Canary Islands. The present study thus expands our knowledge of how the diversity of reproductive traits in islands vs. mainland affects plant mating, establishing new links between reproductive and island biology.

## Results

### *Limonium lobatum* harbors newly discovered diversity of pollen-stigma trait-combinations and is a sexually reproducing species

We combined evidence from pollen and stigma analyses with pollen germination experiments to characterize the diversity of pollen-stigma combinations in *L. lobatum* and determine whether *L. lobatum* reproduces sexually or apomictically.

**Figure 3:**
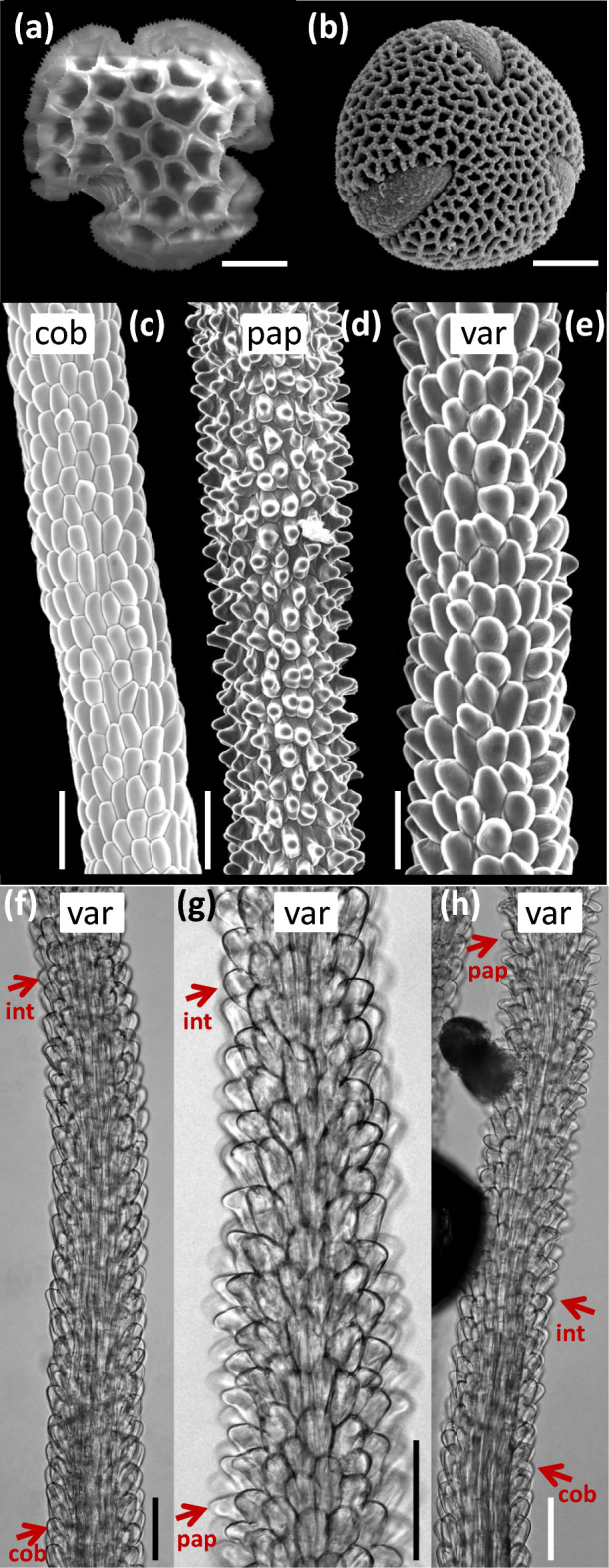
Diversity of pollen exine sculpturing and shapes of stigmatic papillae in *Limonium lobatum*. Previously described pollen types: (**a**) A pollen with coarse exine sculpturing; (**b**) B pollen with fine exine sculpturing. Previously described (typical) stigma types: (**c**) cob stigma with uniformly flat (cob) papillae; (**d**) pap stigma with uniformly protruding, or papillate (pap) papillae. (**e**–**h**) Newly discovered stigma types (var stigmas): (**e**) stigma with all int papillae; (**f**) stigma with cob and int papillae; (**g**) stigma with pap and int papillae; (**h**) stigma with cob, int and pap papillae (see also extended Fig. 3 in Supporting Information). Red arrows illustrate shapes of *cob, pap* and *int* papillae (in red) in var stigmas (**f**–**h**). Stigma types are labelled inside white rectangles (**c**–**h**). Scale bars for (**a**,**b**) indicate 10 µm; scale bars for (**c**–**h**) indicate 50 µm. Photographs (**a**–**e**) were taken with a scanning electron microscope (JEOL 6360 VL; JEOL USA, Inc., Peabody, MA, USA); photographs (**f**–**h**) were taken with an optical microscope (Olympus CH30; Olympus Corporation, Tokyo, Japan) equipped with a digital imaging system (AxioCam; Axio Vision Rel. 4.8; ZEISS, Oberkochen, Germany). For definitions of pollen and stigma traits, see Fig. 4.

**Figure 4:**
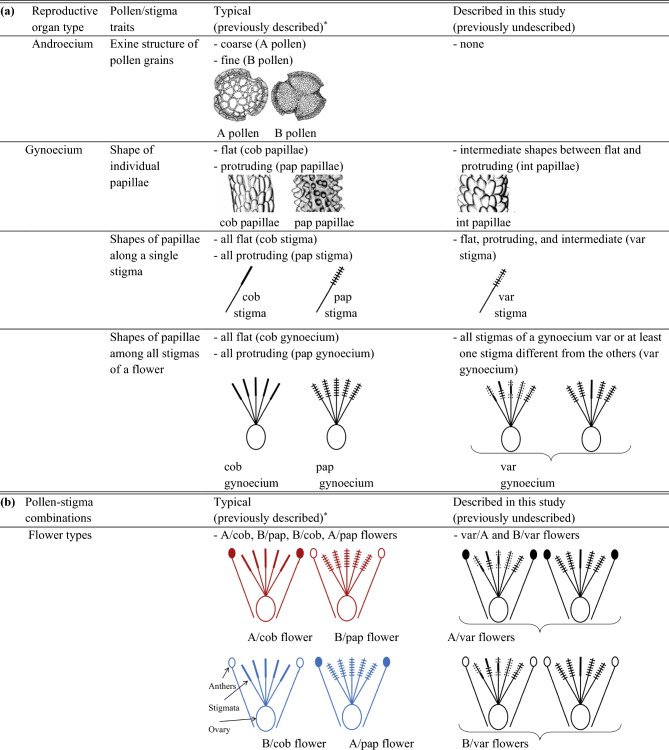

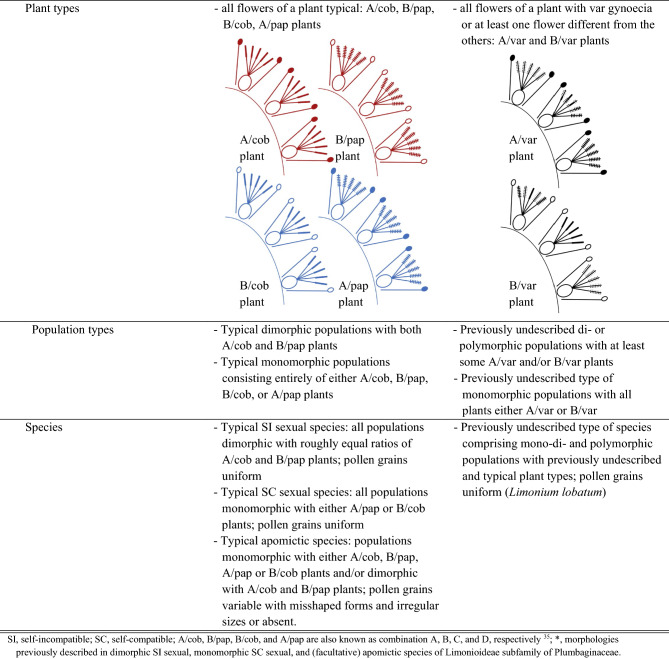
Definitions with corresponding line drawings of typical and newly discovered male and female reproductive traits in *Limonium lobatum*: (**a**) structure of pollen exine (androecium) and shape of stigmatic papillae (gynoecium) within flowers; (**b**) pollen-stigma combinations in hierarchical levels ranging from intra-flower to inter-population. Symbols: Filled symbols represent anthers with A pollen; empty symbols represent anthers with B pollen; absence of cross lines along stigmas indicates flat (cob) papillae; solid cross lines along stigmas indicate protruding, or papillate (pap) papillae; dashed cross lines indicate intermediate (int) shapes of papillae in variable (var) stigmas. Colour coding in (**b**): red and blue, respectively, refer to flowers/plants with pollen-stigma combinations previously defined as SI (A/cob and B/pap) or SC (A/pap and B/cob) in other *Limonieae*; black refers to novel pollen-stigma combinations for which SI or SC were unknown until the present study.

**Figure 5:**
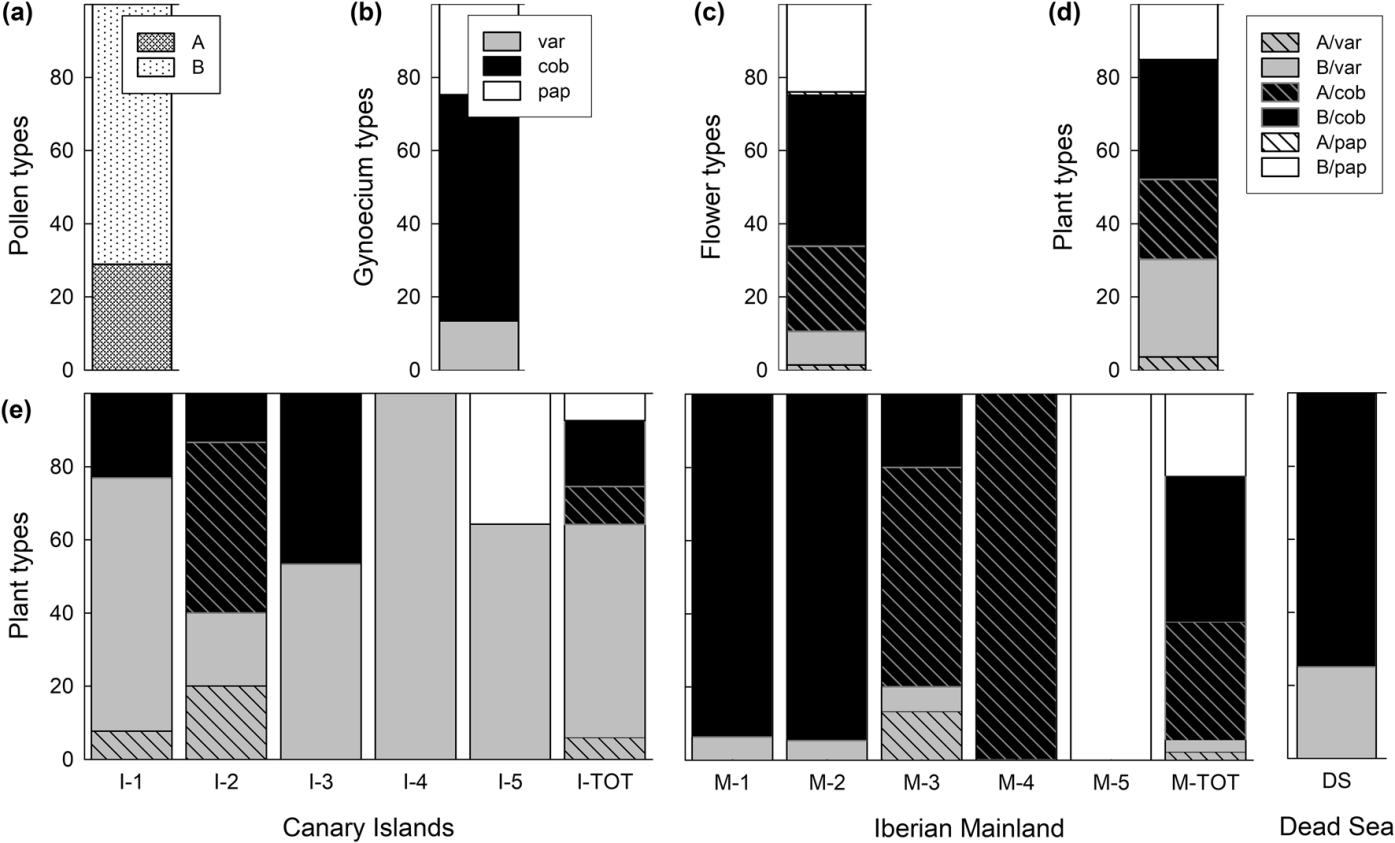
*Limonium lobatum* includes newly discovered diversity of reproductive traits and the proportion of plants with newly discovered stigma-pollen combinations is higher in islands. Upper panels: Proportions of different (**a**) pollen types, (**b**) gynoecium types, (**c**) flower types, and (**d**) plant types across all 165 studied plants. Lower panels: Proportions of different plant types subdivided into five insular (I-1 to I-5), five mainland (M-1 to M-5), and one Dead Sea (DS) populations, with totals for island (I-TOT) and mainland (M-TOT) populations of the Canary Islands and Iberia, showing that islands harbour a larger proportion (64.2%) of plants with newly discovered stigma-pollen combinations than the mainland (5.6%). Location abbreviations as reported in Fig. 2. (**b**–**e**) Symbols: hatched, A pollen; non-hatched, B pollen; black (both hatched and non-hatched), typical morphologies with cob papillae; white (both hatched and non-hatched), typical morphologies with pap papillae; grey, newly discovered type of pollen-stigma dimorphism with var stigmas. Plants with A/pap flowers were not found. For definitions of pollen and stigma traits, see Fig. 4.
  Stigma traits: By examining stigma traits in 2–5 flowers per plant from 203 experimental plants, for a total of 950 analyzed flowers (Supplementary Fig. S3a), we discovered previously unknown types of female reproductive traits at four different hierarchical levels, i.e., papillae within stigmas, stigmas within flowers, gynoecia within plants, and gynoecia among plants. First, at the level of stigma papillae, we discovered a previously undescribed papillae shape that is intermediate between the typical flat (cob) and protruding (pap) shapes: we termed this novel papilla type ‘int papilla’ (Figs. 3 and 4).
  Second, at the level of stigmas, we found that, out of 4723 stigmas, 3020 (64%) had uniformly flat papillae (i.e., typical cob stigmas; Fig. 3c), 1259 (26.6%) had uniformly protruding papillae (i.e., typical pap stigmas; Fig. 3d), and 444 (9.4%) had either uniformly int papillae (Fig. 3e) or comprised various combinations of int, pap, and cob papillae (Fig. 3f–h, Supplementary Table S3): we termed this third, novel stigma-type ‘var stigmas’ (Figs. 3, 4, 5, Extended Fig. 3 in Supplementary Information). Thus, 90.6% of the studied stigmas conformed to previous descriptions (either cob or pap stigmas), while 9.4% of the stigmas (var stigmas) did not.
  Thirdly, at the level of gynoecia, we found that, out of 950 gynoecia, 587 (61.8%) were formed entirely by typical cob stigmas, 235 (or 24.7%) were formed entirely by typical pap stigmas, and 128 (13.5%) were formed by various combinations of var, pap, and cob stigmas (13 gynoecia with cob and pap stigmas; 15 gynoecia with var and cob stigmas; 16 gynoecia with var and pap stigmas; 9 gynoecia with var, pap, and cob stigmas; and 75 gynoecia with all stigmas var): we termed this third, novel gynoecium-type ‘var gynoecia’(Figs. 4, 5b, Supplementary Table S3). Thus, 86.5% of the studied gynoecia conformed to previous descriptions (either cob or pap gynoecia), while 13.5% of the gynoecia (var gynoecia) did not.
  Finally, at the level of plants, we found that, out of 203 plants, 101 (49.8%) had flowers with typical cob gynoecia, 29 (14.2%) had flowers with typical pap gynoecia, and 73 plants (36.0%) had flowers with various combinations of var, pap, and cob gynoecia (10 plants with cob and pap gynoecia; 22 plants with var and cob gynoecia; 15 plants with var and pap gynoecia; 23 plants with var, cob, and pap gynoecia; and 3 plants with all flowers var): we termed this novel plant-type ‘var plants’. Thus, 64.0% of the studied plants conformed to previous descriptions (either cob or pap plants), while 36.0% of the plants (var plants) did not. To summarize, newly discovered types of stigma traits occurred both within and among flowers of individual plants and among plants (Fig. 4).
  Pollen-stigma combinations: By combining the newly generated knowledge of pollen and stigma traits reported above, we discovered previously undescribed pollen-stigma combinations within flowers, among flowers of the same plant, and among plants of the same population (Figs. 4, 5).
  At the flower level (“Flower types” in Fig. 4), out of 768 flowers, 687 (89.5%) had typical pollen-stigma combinations previously regarded as being associated with either SI (A/cob: 179 flowers; B/pap:184 flowers) or SC (B/cob: 317 flowers; A/pap: 7 flowers) and 81 (10.5%) had previously undescribed pollen-stigma combinations (A/var: 11 flowers; B/var: 70 flowers; Fig. 5c, Supplementary Table S3) that, consequently, could not be defined as either SI or SC based on morphology.
  At the plant level (“Plant types” in Fig. 4), out of 165 plants, 115 (69.7%) had flowers with typical pollen-stigma combinations (36 A/cob plants and 25 B/pap plants, both previously thought of being SI; 54 B/cob plants, previously thought of being SC), while A/pap plants were not found. Additionally, we discovered that 50 (30.3%) of 165 plants comprised flowers with newly described types of pollen-stigma combinations (6 A/var plants with variable combinations of A/var, A/cob, and A/pap flowers; 44 B/var plants with variable combinations of B/var*,* B/cob, and B/pap flowers; Fig. 5d, Supplementary Table S3).
  At the population level (“Population types” in Fig. 4; see also Fig. 5e), none of the 11 analyzed populations was typically dimorphic (i.e., comprising exclusively A/cob and B/pap plants) and none was typically monomorphic (i.e., consisting of either B/cob or A/pap plants). Furthermore, plant types were distributed across the 11 populations as follows (see also Supplementary Fig. S3 for number of plants per population analyzed for pollen and stigma traits). Only three populations were monomorphic: I-4 (10 plants, all newly described B/var type); M-4 (20 plants, all A/cob type, previously thought to be SI), and M-5 (20 plants, all B/pap type, previously thought to be SI). Five populations were dimorphic with previously described and newly discovered plant types: I-3 (15 plants, of which 7 B/cob and 8 B/var), I-5 (14 plants, of which 5 B/pap and 9 B/var); M-1 (16 plants, of which 15 B/cob and 1 B/var); M-2 (19 plants, of which 18 B/cob and 1 B/var) and DS (8 plants, of which 6 B/cob and 2 B/var). Three populations were polymorphic with both previously described and newly discovered plant types: I-1 (13 plants, of which 3 B/cob, 9 B/var, and 1 A/var); I-2 (15 plants, of which 2 B/cob, 7 A/cob, 3 B/var, and 3A/var); and M-3 (15 plants, of which 3 B/cob, 9 A/cob, 1 B/var, and 2 A/var; Fig. 5e, Supplementary Table S3).
(ii) *Pollen-germination experiments in monomorphic populations *Analyses of pollen germination and pollen-tube growth in flowers from the three monomorphic populations identified above (I-4, M-4, M-5) revealed that A pollen adhered, germinated, and formed pollen tubes that penetrated cob stigmas in M-4 plants, and *B* pollen adhered, germinated, and formed pollen tubes that penetrated both var and pap stigmas in I-4 and M-5, respectively (Supplementary Fig. S4; results of seed-set are reported in section B below). Furthermore, pollen grains were consistently well-developed and homogeneous in size. Both results imply that plants in monomorphic populations were SC and support the conclusion that *L. lobatum* reproduces sexually.

(i) *Pollen and stigma analyses* Pollen traits: Pollen grains of 567 flowers from 167 experimental plants (Supplementary Fig. S3a) were generally well developed, homogeneous in size, and had either coarse (A pollen: 164 out of 567 flowers, or 28.9%) or fine exine sculpturing (B pollen: 403 out of 567 flowers, or 71.1%; Figs. 3, 4, and 5a). Moreover, each of the 140 plants from which multiple flowers were analyzed had either A pollen (i.e., 38 plants, or 27.1%) or B pollen (i.e., 102 plants, or 72.9%) (Supplementary Table S3), warranting the conclusions that pollen-type (either A or B) is stable within plant, a property useful to determine pollen-stigma trait-combinations (see below), and *L. lobatum* reproduces sexually.

### *Limonium lobatum* is mostly SC and can reproduce uniparentally

Due to the death of some plants initially included in the pollination experiments and limited flower production of other plants, seed-set (for definition, see “Methods”) was tallied for 2881 flowers from a total of 174 experimental plants (1633 flowers from 99 island plants, divided in 24 previously described, 64 newly discovered, and 11 undetermined plant types; and 1248 flowers from 75 mainland plants, divided in 63 previously described, five newly discovered, and seven undetermined plant types; Supplementary Fig. S3b and Supplementary Table S4). The undetermined plant types were included in analyses aimed at testing Baker’s law (see section C below), but excluded from analyses comparing seed-set among plant types. Seed-set was used to compare island and mainland plants for capacity of uniparental reproduction. All formed seeds were well developed and homogeneous in size.

**Figure 6:**
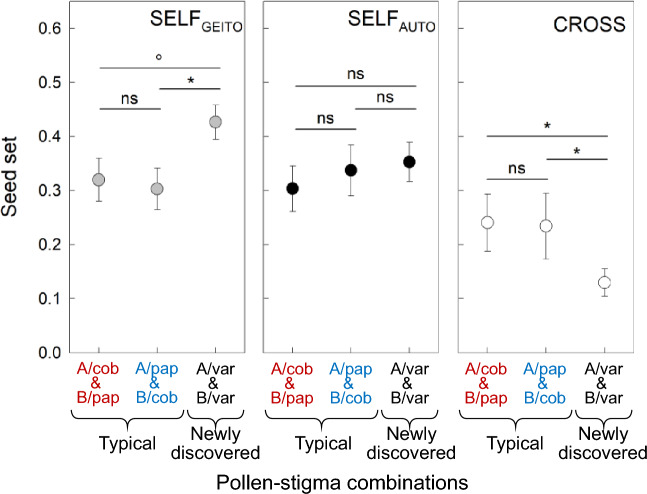
Comparison of seed-set after manual selfing and crossing experiments in plants with newly discovered pollen-stigma combinations (A/var and B/var) vs. plants with typical pollen-stigma combinations subdivided into combinations previously thought to be SI (A/cob and B/pap: red) and SC (A/pap and B/cob: blue) in *Limonium lobatum*. Abbreviations: SELF_AUTO_, autonomous selfing; SELF_GEITO_, geitonogamous selfing; and CROSS, cross-pollination. Significance levels: *P ≤ 0.05, °*P* = 0.067, and ns *P* > 0.067. Sequential Bonferroni correction was used to account for multiple tests. Error bars denote standard errors, SE. For definitions of stigma and pollen traits, see Fig. 4.

Only 45 (28 previously described, 12 newly discovered, and 5 undetermined plant types) of 164 plants (~ 27%) had a geitonogamous seed-set of 0–20% (Supplementary Table S4), implying that these plants had a fully to partially functional SI system. The remaining 119 (52 previously described, 54 newly discovered, and 13 undetermined plant types) of the 164 plants studied (~ 73%) had a geitonogamous seed-set of 20–100%, implying that these plants had a weakened SI system, hence can be considered SC (results of pollen-germination are reported in section A-*ii* above)^53,54^. Surprisingly, we found that in plants with previously described pollen-stigma combinations, both selfed and outcrossed seed-set did not significantly differ between plant types with pollen-stigma combinations typically regarded as SI (A/cob and B/pap) vs. those typically regarded as SC (B/cob or A/pap; SELF_AUTO_: *t* = − 0.214, d.f. = 423, *P* = 1.000; SELF_GEITO_: *t* = 0.328, d.f. = 211, *P* = 0.743; CROSS: *t* = 0.659, d.f. = 1030, *P* = 0.510; Fig. 6; for detailed results of the generalized linear mixed effects model [GLMM] see Supplementary Table S2a and Supplementary Fig. S5a). Moreover, seed-set after autonomous selfing (SELF_AUTO_) did not significantly differ among the three plant types, namely plants with previously described pollen-stigma combinations thought to be SI (A/cob or pap/B), plants with previously described pollen-stigma combinations thought to be SC (A/pap or B/cob), and plants with newly described pollen-stigma combinations (A/var or B/var) of unknown pollen compatibility (-0.214 ≥ *t* ≤ 0.788; 394 ≥ d.f. ≤ 423; *P* = 1.000; Fig. 6). Importantly, plants with newly described pollen-stigma combinations had (significantly) higher seed-set after manual selfing and significantly lower seed-set after manual crossing than plants with previously described pollen-stigma combinations, whether they were expected to be SI (SELF_GEITO_:* t* = 2.139, d.f. = 195, *P* = 0.067; CROSS: *t* = − 2.469, d.f. = 1269, *P* = 0.040) or SC (SELF_GEITO_: *t* = 2.593, d.f. = 201, *P* = 0.031; CROSS: *t* = − 2.480, d.f. = 786, *P* = 0.040; see Fig. 6).

### Newly discovered plant types and capacity for uniparental reproduction are enriched on islands

**Figure 7:**
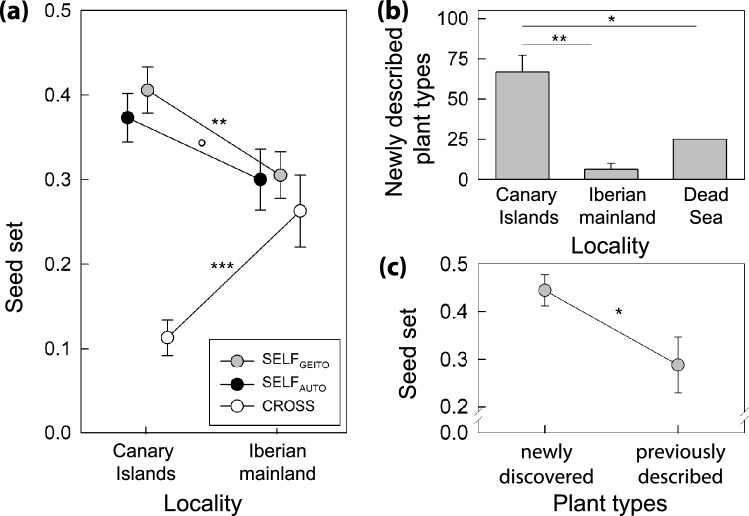
Island plants have higher capacity for uniparental seed production and plants with newly discovered pollen-stigma combinations produce more selfed seed than plants with typical pollen-stigma combinations on islands. (**a**) Seed-set after manual selfing and crossing experiments: SELF_AUTO_, seed-set after autonomous selfing; SELF_GEITO_, seed-set from manual crosses between flowers of the same plant (geitonogamous selfing); CROSS, seed-set from manual crosses between flowers of different plants. (**b**) Comparative proportions of newly described plant types (A/var and B/var) of *L. lobatum* in Canary Islands, Iberian mainland, and Dead Sea localities. (**c**) Seed-set of insular plants after manual selfing (SELF_GEITO_) subdivided into plants with newly discovered pollen-stigma combinations (A/var and B/var) vs. plants with previously described pollen-stigma combinations (A/cob, B/pap, A/pap, B/cob). Significance levels: ***P ≤ 0.001, **P ≤ 0.01, *P ≤ 0.05, °*P* = 0.068. Error bars denote standard errors SE.

(i) *Island plants have higher capacity for uniparental reproduction than mainland plants (test of Baker’s law) *Island plants had (significantly) higher seed-set after self-pollination (SELF_GEITO_:* t* = 2.601, d.f. = 222, *P* = 0.010; SELF_AUTO_:* t* = 1.827, d.f. = 462, *P* = 0.068) and significantly lower seed-set after cross-pollination than mainland plants (CROSS: *t* = − 4.705, d.f. = 805, *P* ≤ 0.001; Fig. 7a; for detailed GLMM results see Supplementary Table S2b and Supplementary Fig. S5b).
(ii) *More plants with newly discovered pollen-stigma combinations occur on islands than the mainland *Plants with newly discovered pollen-stigma combinations were overrepresented on the Canary Islands (43 novel plant types vs. 24 typical plant types) vs. the geographically closer Iberian mainland (5 novel plant types vs. 85 typical plant types; one-tailed *t*-test: *t* = 5.557, d.f. = 8, *P* < 0.001) and the geographically distant Dead Sea shore (2 novel plant types vs. 6 typical plant types; one-tailed, one-sample *t*-test: *t* = 4.080, d.f. = 4, *P* = 0.008; Figs. 5e, 7b).
(iii) *Island plants with newly discovered pollen-stigma combinations have higher capacity for uniparental reproduction than island plants with previously described pollen-stigma combinations *Insular plants with newly discovered pollen-stigma combinations produced significantly more seeds when manually selfed (SELF_GEITO_) than insular plants with previously described pollen-stigma combinations (*F* = 6.276; d.f. = 1,76; *P* = 0.014; Fig. 7c; for detailed GLMM results see Supplementary Table S2c and Supplementary Fig. S5c). Therefore, the enriched capacity for uniparental reproduction on islands can be attributed to the increased capacity of newly discovered plant types to produce selfed seed.

## Discussion

Our results demonstrate that *L. lobatum* does not conform to previous descriptions as a pollen-stigma dimorphic, hence presumed SI species^14^ ; rather, it harbors combinations of reproductive traits documented for the first time here. First, we discovered an uneven proportion of plants with either A (27.1%) or B (72.9%) pollen grains that does not conform to the expectation of isoplethy typical of species with pollen-stigma dimorphism. Secondly, we discovered previously undescribed types of stigma papillae and pollen-stigma combinations (Figs. 3, 4). Specifically, we found a new type of stigma papillae with a shape that is intermediate (int) between the protruding (pap) and flat (cob) shapes of the stigma papillae previously described in typical pollen-stigma dimorphic species^14,16,37^. The combination of pollen and stigma traits enabled the assignment of *L. lobatum* plants to five discrete categories, namely (i) plants comprising exclusively A/cob flowers; (ii) plants comprising exclusively B/pap flowers; (iii) plants comprising exclusively B/cob flowers; (iv) plants comprising A/var flowers with variable combinations of cob, pap, and int papillae; and (v) plants comprising B/var flowers with variable combinations of cob, pap, and int papillae. Plants with typical A/pap flowers were not found (Fig. 5). The first three plant types match typical descriptions of pollen-stigma dimorphic (i, ii) or monomorphic (iii) species. The last two types (iv, v) do not match previous descriptions and are described for the first time here. To summarize, the key difference between plants with newly described pollen-stigma combinations (i.e., var plants: 30.3%) and typical plants (69.7%) is that the flowers of the former include papillae of different shapes, while the flowers of the latter comprise exclusively either cob (cob plants) or pap papillae (pap plants), hence have consistent papillae shapes (Figs. 1, 3c–h, and 4).

Furthermore, we experimentally demonstrated that, contrary to previous descriptions of *L. lobatum* as a pollen-stigma dimorphic, hence presumed self-incompatible species (Baker^14^), ≥ 73% of all plants were capable of setting seeds uniparentally when their flowers were manually self-pollinated (SELF_GEITO_ in Supplementary Table S4). Indeed, a surprising discovery was that A pollen adhered and formed pollen tubes in cob stigmas and B pollen adhered and formed pollen tubes in pap stigmas, two pollen-stigma combinations previously thought to be SI (Supplementary Fig. S4). Thus, our results imply that the previously established correspondence between specific pollen-stigma combinations and SI or SC, possibly relying on the hypothesis of topological complementarity^32,33^, does not apply to *L. lobatum*. This is the first time that such a lack of correspondence has been described except for a single population in the Shetland Islands^6^ and a few populations growing on metal-contaminated soils^30,45,55^ in the exceptionally variable species complex of *Armeria maritima*^56,57^.

Overall, results from seed-set (Figs. 6 and 7a; Supplementary Table S4) suggest a weakening of the SI system in *L. lobatum*. Moreover, plants with newly described pollen-stigma combinations (i.e., var plants), which include flowers with different papillae shapes (cob, pap, and int) in their gynoecia, produce higher seed-set after selfing than plants with uniform papillae type (either cob or pap plants; Fig. 6). Therefore, plants with newly discovered pollen-stigma combinations have an even weaker SI system, hence higher capability to produce seeds uniparentally and lower capability to produce seeds biparentally than plants with previously described reproductive morphologies (Fig. 6). Altogether, our results support the conclusion that *L. lobatum* harbors newly discovered pollen-stigma combinations and a weaker SI system than previously assumed, hence it represents a previously unknown type of species comprising mono-, di-, and polymorphic populations with both typical and novel plant types for pollen-stigma combinations capable of forming uniparental seeds (Figs. 5d,e, 6). Since these morphological traits are first reported here, no previous knowledge about their inheritance patterns are available. Future crossing experiments will be necessary to determine the inheritance and better assess the evolutionary implications of the reproductive combinations first documented here.

To summarize, our findings demonstrate that the newly discovered plant types of *L. lobatum* have a higher capacity for uniparental seed production than previously described plant types (Fig. 6), are overrepresented in the Canary Islands vs. the mainland (Fig. 7b), and disproportionally contribute to the enriched capacity for uniparental seed production on islands (Fig. 7c). Thus, our results link the enrichment of newly discovered plant types and uniparental reproduction on islands, supporting Baker’s law.

### *Limonium lobatum* is a sexually reproducing species harboring newly discovered pollen-stigma combinations, especially on islands

The production of seeds from a single plant, at the core of Baker’s law, can occur sexually via self-fertilization, regardless of whether it is aided by pollinators or not, and/or asexually via vegetative propagation or apomixis. Since *L. lobatum* is an annual species, vegetative propagation can be excluded. Therefore, we proceeded to test whether *L. lobatum* reproduces apomictically, a conclusion that would be supported if: (i) pollen grains were absent or, if present, misshaped^14,35^; and (ii) plants in the three monomorphic populations turned out to be SI, implying seed production via apomixis^14,32,35^. We found that pollen grains of *L. lobatum* were consistently well developed and homogeneous in size (Figs. 3a,b, 5a) and cob and pap stigmas were compatible with A and B pollen, respectively, in monomorphic populations (Supplementary Fig. S4), suggesting that this species reproduces sexually rather than apomictically. Previous studies lend additional support to the conclusion that *L. lobatum* is not apomictic: (i) apomixis is usually associated with polyploidy, while being extremely rare in diploid, annual species, such as *L. lobatum*^25^; (ii) all apomictic species described in *Limonium* belong to *Limonium* subg*. Limonium,* a clade phylogenetically distinct from *Limonium* subg*. Pteroclados*, which includes *L. lobatum*^29,58^. Thus, all available evidence supports the conclusion that *L. lobatum* is a sexually reproducing species that can produce seeds uniparentally only via self-fertilization but not via apomixis.

In *Limonieae*, sexually reproducing taxa typically consist of pollen-stigma dimorphic populations (i.e., comprising only A/cob and B/pap plants, typically SI) or pollen-stigma monomorphic populations (i.e., comprising either B/cob or A/pap plants, typically SC; Fig. 1)^6,14–16,30^. However, none of the populations of *L. lobatum* was typically dimorphic or typically monomorphic (Fig. 5). Rather, we found previously undescribed types of pollen-stigma combinations among flowers within plants, among plants of the same population, among populations, and an enrichment of plants with newly discovered pollen-stigma combinations on islands (Figs. 5, 7b). Further, since most plants were capable of setting seeds uniparentally when they were manually self-pollinated (Figs. 6, 7a, and Supplementary Table S4), all lines of evidence suggest that *L. lobatum* is a sexually reproducing, largely SC species characterized by novel diversity of reproductive traits never documented before in *Plumbaginaceae* nor, to the best of our knowledge, in other systems described as pollen-stigma dimorphic or monomorphic^14,16,31^.

Indeed, deviations from typical surface morphologies of pollen (coarse vs. fine exine sculpturing) or stigmas (flat vs. protruding papillae cells) are rare in *Limonieae*. Such deviations were noticed, to our knowledge, only in *L. humile* and *Armeria maritima* subsp. *sibirica*^32,59–61^. Specifically, pollen grains with variable or intermediate exine sculpturing and stigmas with homogeneously intermediate papillae shapes were found in these two taxa, although the latter was attributed to premature termination of stigma development due to self-pollination in still-closed flowers of *A. maritima* subsp. *sibirica*^32,59,60^. Conversely, in *L. lobatum* we found no variability of pollen morphology but variability of female reproductive structures not attributable to immature stage of floral development. Indeed, we documented well-developed stigmas characterized by papillae of flat (cob), protruding (pap) and/or intermediate (int) shapes, allowing us to describe a new stigma type (i.e., var stigma; Figs. 3, 4). Moreover, papillae shapes differed among stigmas of a gynoecium and among flowers of a plant, revealing levels of diversity never documented before in pollen-stigma dimorphic systems (Figs. 3, 4, 5, Extended Fig. 3 in Supplementary Information). The discovery that female reproductive structures can differ within plants, while male reproductive structures remain homogeneous, may reflect the fact that flowers are developmental and functional mosaics comprising four organ whorls (calyx, corolla, androecium, and gynoecium), each with its own developmental genetic regulation^62,63^.

What are the potential causes of the unexpected diversity of female reproductive traits we discovered in *L. lobatum*, especially in island plants? Variability of reproductive structures can be caused by developmental stage or position in an inflorescence, developmental plasticity, and/or developmental instability^62–65^. Developmental plasticity tends to produce consistent patterns of trait variation^64,65^, while developmental instability tends to produce more irregular patterns of trait variation^62–64^. The latter phenomenon might occur in plants of *L. lobatum* characterized by previously undescribed diversity of female traits, where the shapes of stigmatic papillae differed among stigmas of individual flowers and flowers of individual plants without any discernible, regular patterns, especially in islands (Figs. 3, 4, 5). The inconsistent pattern of papillae shapes may be caused by homozygosity, which can introduce random errors during organ development^64^. Since selfing increases homozygosity, the floral organs of SC plants may exhibit elevated developmental instability variation^62–64^.

Islands harbored more plants of *L. lobatum* with newly described pollen-stigma combinations (Figs. 5e, 7b) and higher capacity for uniparental reproduction than the mainland (Fig. 7a), implying that selfing is elevated in islands, especially in plants with newly discovered pollen-stigma combinations (Fig. 7c). Therefore, our results are congruent with the proposed notion that developmental instability is particularly pronounced in isolated, peripheral, self-compatible populations of both animals and plants that are more likely to self-fertilize^66^. Although developmental instability is often assumed to be maladaptive, intra-plant diversity can also have adaptive potential^66–71^. This may be the case, for example, when it affects reproductive traits that enable self-pollination, as demonstrated in *Eichhornia paniculata*, where a high level of developmental instability in style elongation promoted self-pollination in plants growing in harsh environmental conditions^69,70^. In *L. lobatum*, novel combinations of pollen-stigma traits and higher capacity for uniparental reproduction are connected, especially on islands (Figs. 6, 7). Future studies are required to test whether the increased ability to self-fertilize detected in plants with newly described pollen-stigma combinations indeed provides reproductive assurance, hence may be adaptive, in natural island populations.

### The capacity for uniparental reproduction is enriched in island populations of *Limonium lobatum*

The number of SC individuals in a population should increase when the selective advantages of self-fertilization outweigh its costs^72–74^. Since the ability for self-fertilization may provide reproductive assurance when mates are limited^8^, selection should favor SC, hence the capability for uniparental reproduction, in annual plants and drive the enrichment of SC in islands^21,75–78^. Corroborating the first prediction, we found that most plants of the annual *L. lobatum* were able to set seeds when they were manually self-pollinated (Figs. 6, 7a; Supplementary Table S4) and, more specifically, plants with newly discovered pollen-stigma combinations had higher seed-set after self-pollination treatments than plants with previously described traits (Fig. 6). Furthermore, the proportion of plants with newly discovered pollen-stigma combinations was higher in islands than in the mainland (Figs. 5e, 7b). The occurrence of plants that can set seeds uniparentally in mainland populations (Fig. 7a) likely enabled the colonization of islands after long-distance dispersal by seeds that produced plants able to reproduce without mates. Since only SC plants can set seed uniparentally, such plants were overrepresented on islands, corroborating the prediction that selection should favor the enrichment of SC plants in islands and supporting Baker’s law (Fig. 7a). Furthermore, plants with newly discovered pollen-stigma combinations disproportionally contributed to the enriched capacity for uniparental seed production on islands (Fig. 7c). In summary, island plants, particularly the ones with previously undescribed pollen-stigma combinations, displayed an increased ability for uniparental reproduction. Hence, our results fit the expectations of Baker’s law that individuals from mainland populations with the capability for uniparental reproduction are more likely to colonize and become established in islands after long-distance dispersal, resulting in the enrichment for uniparental reproduction on islands.

The transition from outcrossing to selfing is a two-step process^79^: (i) a shift from SI to SC at the individual level when mutations weaken/disable the SI system in individual plants; (ii) a shift to inbreeding at the population level when selection favors individuals with a weakened/disabled SI system, resulting in the spread and fixation of the selfing allele within a population. The latter usually occurs only when inbreeding depression is weak (e.g., loci with recessive deleterious mutations are rare in a population), which is often expected after genetic purging due to strong selection for selfing in early phases of colonization^11,77,80^. Selfed plants of *L. lobatum* produced as many or more seeds than outcrossed plants in both islands and mainland (Fig. 7a), suggesting that inbreeding depression is absent or negligible at the stage of seed production in *L. lobatum*. This result is consistent with findings in predominantly self-fertilizing populations^81^ and species^80^ reporting the absence of inbreeding depression at the seed-production stage. Since inbreeding depression can be expressed at later stages of the life cycle^82^, we cannot exclude the possibility that it could affect the fitness of selfed *L. lobatum* plants.

Moreover, selfed and outcrossed seed-sets were similar on the mainland, whereas selfed seed-set was significantly higher than outcrossed seed-set on islands (Fig. 7a). This finding suggests that in insular populations of *L. lobatum* genetic incompatibilities might hinder the formation of outcrossed seeds (i.e., outbreeding depression)^83–85^. Genetic factors known to cause outbreeding depression include underdominant loci, chromosomal rearrangements, and negative epistatic interactions, for instance of alternative alleles at different loci^86,87^. Selfing increases the probability of fixation of such genetic factors by reducing genetically effective recombination, increasing linkage disequilibrium within populations, and exacerbating epistatic interactions across the genome^2,87–90^. Consequently, within-population outbreeding depression has only been reported in populations with considerable levels of selfing^83,84,86,87,91–98^. It is therefore likely that island plants of *L. lobatum* self-fertilize more frequently than mainland plants and suffer from outbreeding depression. Indeed, SC species may experience higher levels of selfing in islands vs. mainland populations if pollinator services are unreliable and mates scarcer on islands^11^. Our experiments established that *L. lobatum* can set seeds uniparentally and that uniparental reproduction is enriched on islands vs. mainland, a result central to supporting Baker’s law (Fig. 7a). Future field studies, crossing experiments, and population genomic analyses should investigate the extent of autonomous vs. pollinator-assisted selfing-rates and the potential causes and genetic basis of outbreeding depression in island populations of *L. lobatum*.

## Conclusions

Contrary to previously published accounts, we discovered that *L. lobatum* is a largely SC species characterized by previously unknown pollen-stigma combinations. Plants with newly described pollen-stigma combinations which had higher seed-set after self-pollination treatments than typical plants and were overrepresented on islands, reflecting the enrichment of plants capable of uniparental reproduction on islands predicted by Baker’s law^12^. Additionally, we documented that selfed seed-set was significantly higher than outcrossed seed-set on islands, even though outcrossed and selfed seed-set were similar on the mainland, suggesting that genetic incompatibilities might hinder the formation of outcrossed seeds in island plants. Conformant with the predictions of Baker’s law, our study supports the notion that individuals with a higher capacity for uniparental reproduction are more likely to colonize distant, isolated habitats. Furthermore, while the genetic basis of pollen-stigma dimorphism remains unknown, the knowledge generated in this study—especially the discovery of the skewed proportions of plants with A and B pollen, with a preponderance of the latter, and new combinations of pollen and stigma traits—provides a useful foundation for future studies aimed at elucidating the genes controlling pollen-stigma dimorphism. In conclusion, our results confirm that comparative trait-based approaches of island and mainland populations are well-suited to elucidate the eco-evolutionary dynamics of long-distance colonization, providing new links between reproductive and island biology.

## Methods

### Seed collection, germination, and cultivation of experimental plants

In autumn 2011, we collected seeds from each of 20 randomly selected plants (i.e., seed parents) in each of five natural populations in the Canary Islands (I-1 in Tenerife and I-2, I-3, I-4, I-5 in Fuerteventura) and five populations in the southern part of the Iberian mainland (M-1, M-2, M-3, M-4, M-5; Fig. 2, Supplementary Fig. S3). All necessary permits were obtained to collect seeds of *Limonium lobatum* in Spain and the Canary Islands, hence the collection of seeds complies with all relevant institutional, national, and international guidelines and legislation. Plants were taxonomically identified by Ares Jiménez and one voucher specimen per population is kept at Herbarium Z (Zürich, Switzerland; Supplementary Table S1). Additionally, we received seeds from a natural population on the shores of the Dead Sea (DS) via the Jerusalem Botanical Gardens (Israel; IL0Z-20150596).

In spring 2012, we germinated 10 seeds from each of the original 20 seed parents per population in islands (I-1 to I-5) and mainland (M-1 to M-5), resulting in 200 seedlings per population (for a total of 1000 seedlings across the ten populations); we then randomly transplanted 20 seedlings per seed parent per population into individual pots (for a total of 200 seedlings), thus each plant originally sampled from the natural populations was represented in the plants used in subsequent pollen-transfer experiments performed in the greenhouse. We also transplanted 10 seedlings from seeds of the Dead Sea population, for a grand total of 210 seedlings used in subsequent experiments (Supplementary Fig. S3). All seed germinations and seedling transplants were performed on the same day. Experimental plants were cultivated under standard conditions in the pollinator-free environment of a greenhouse at the Botanical Garden of the University of Zürich, Switzerland (temperature: 18–22 °C; humidity: ~ 65%; photoperiod: 12 h dark /12 h light, minimum 30,000 LUX). All plants started blooming 10–12 weeks after germination. For sampling and experimental design used to characterize pollen and stigma traits and seed-set in *L. lobatum*, see Supplementary Fig. S3.

### Is *L. lobatum* a typical pollen-stigma dimorphic, sexually reproducing species?

(i) *Pollen and stigma analyses *To test whether *L. lobatum* represents a typical pollen-stigma dimorphic species (as reported by Baker^14^) or not (as suggested by preliminary morphological observations in the field by A. Jimenez, pers. obs.), we analyzed pollen and stigma traits. The observed pollen and stigma traits and their combinations were then compared with previously described morphologies to determine whether pollen grains and stigma-papillae shapes matched previous descriptions and were both equally distributed between A and B pollen and cob and pap stigmas, conforming to the expectations of isoplethy in a pollen-stigma dimorphic species, as assumed by Baker^14^.
  Reproductive traits can vary at different levels, i.e., within the androecium or gynoecium of a single flower, among flowers within plants, among plants of the same population, and among plants of different populations^62–65,99^. Therefore, we checked for consistency of pollen and stigma traits among flowers within plants by analyzing 2–5 flowers per plant (see below for sampling details) and excluded from subsequent analyses of stigma traits five plants from which we had harvested only one flower (for pollen traits, see below). Out of the flowers initially harvested for morphological analyses, 392 had already shed all pollen from the anthers and two had shriveled gynoecia, thus such reproductive organs could not be utilized for subsequent analyses. Flowers were harvested on the same day across plants whenever possible to minimize potential developmental and positional effects on subsequent morphological analyses (see also Methods and Results S1 in Supplementary Information)^62,63^. Anthers and stigmas were covered with a drop of water on a microscope slide and examined at 40 × magnification using an optical light microscope (Olympus CH30; Olympus Corporation, Tokyo, Japan) equipped with a digital imaging system (AxioCam; Axio Vision Rel. 4.8; ZEISS, Oberkochen, Germany).
  *Pollen traits* - We recorded the coarseness of exine sculpturing in the pollen grains of 567 flowers from 167 plants (Supplementary Fig. S5a; Supplementary Table S3a). Examination of 140 plants from which 2–5 flowers each were analyzed showed that pollen traits of *L. lobatum* did not differ among flowers within plant. Therefore, it was possible to assign all flowers of each plant to a single pollen type as long as at least one flower in a plant had pollen-filled anthers, which was the case for all 167 plants analyzed for pollen traits.
  *Stigma traits* - We recorded the shapes of papillae cells along linear stigmas in the gynoecium of 2–5 flowers per plant from 203 plants, for a total of 4723 stigmas analyzed in 950 flowers.
  *Pollen-stigma combinations -* Given the independent assessment of pollen traits in 167 plants and gynoecium type in 203 plants, it was possible to determine pollen-stigma combinations in 768 flowers of 165 plants.
  The pollen and stigma analyses above enabled us to assign each analyzed plant from the 11 sampled populations (Fig. 2) to either previously-described (i.e., A/cob, B/pap, thought to be SI; and B/cob, A/pap, thought to be SC) or newly-discovered categories of pollen-stigma combinations (i.e., A/var and B/var, with unknown pollen compatibility) and plant types (see Figs. 3, 4 for photos, definitions, and line drawings of pollen-stigma combinations and plant types and Fig. 5e for distributions of plant types in island and mainland populations). The knowledge generated above (see “Results”) set the stage for the pollen-germination and seed-set experiments below.
(ii) *Pollen-germination experiments in monomorphic populations* As explained above, *L. lobatum* is a diploid annual, excluding clonal propagation and implying reproduction via seed, either sexually or apomictically. In monomorphic populations (i.e., I-4, M-4; M-5; see Results and Fig. 5e), sexual seed production can only occur if plants are self and/or intra-morph compatible. Through manual pollen-transfer and germination experiments, we thus tested whether 16 plants from the three monomorphic populations above were SC, hence likely to produce seeds sexually, or SI, hence likely to produce seeds apomictically. Expectations were mixed: for I-4, no expectations were possible, since the population consisted exclusively of newly discovered types of plants (B/var; see “Results” and Fig. 4) with unknown pollen compatibility; for M-4 and M-5, expectations were conflicting, because both populations were monomorphic, hence expected to comprise SC plants, but plants were either A/cob in M-4 or B/pap in M-5, both pollen-stigma combinations previously regarded as SI (see “Results” and Figs. 4, 5).
  We then proceeded to screen pollen grains for morphological irregularities (which would be suggestive of apomictic reproduction if plants were SI) and tested 16 plants for pollen compatibility by manually selfing six B/var flowers of four plants from I-4, ten A/cob flowers of five plants from M-4, and 15 B/pap flowers of seven plants from M-5. Flowers were harvested 24–48 h after pollination, dissected, and stigmas stored in 70% ethanol. Pollen tubes were stained and microscope slides prepared following Martin’s^100^ method with minor adjustments. For each experimental flower, we visually checked whether any of the applied pollen grains germinated and any of the budding pollen tubes penetrated the stigmatic surface by direct illumination with UV-light using an optical microscope (Olympus Bx50; Olympus Corporation, Tokyo, Japan) equipped with a digital imaging system (AxioCam; Axio Vision Rel. 4.8; ZEISS, Oberkochen, Germany).

### Can *L. lobatum* produce seeds uniparentally?

To assess whether *L. lobatum* can produce seeds uniparentally, we estimated seed-set. Since each *L. lobatum* flower has only one ovule (Supplementary Fig. S2) and can produce only one seed, seed-set in *L. lobatum* is defined as the proportion of flowers per plant that produce a seed (note that seed-set and fruit-set are the same in *L. lobatum*). Controlled pollination experiments were performed on a total of 180 experimental plants (20 plants per population from all five Canarian populations for a total of 100 plants, divided in 23 previously described, 66 newly discovered, and 11 undetermined plant types; and 20 plants per population from four Iberian populations for a total of 80 plants, divided in 65 previously described, six newly discovered, and nine undetermined plant types; M-5 and DS were excluded from the experiment, as they did not produce enough flowers; Supplementary Fig. S3b). Undetermined plant types were included in analyses addressing the question of whether *L. lobatum* can produce seeds uniparentally and testing of Baker’s law, but excluded from analyses comparing seed-set among plant types. We used intact flowers, since it was not possible to remove the anthers without transferring considerable amounts of self-pollen to the stigmas, as buds of *L. lobatum* are small and anthers dehisce when flowers open (B. Alther, pers. observation; see also^33^). All plants were individually caged to exclude pollinators. In freshly opened, intact flowers of each plant, we executed three treatments: (a) no pollen was manually transferred onto stigmas, allowing only for autonomous fertilization within the same flower (SELF_AUTO_ treatment); (b) stigmas were manually pollinated with pollen from two flowers of the same plant, allowing for both geitonogamous and autonomous self-fertilization (SELF_GEITO_ treatment); and (c) stigmas were cross-pollinated with pollen from two flowers of two randomly selected plants from the same population, allowing for both cross-fertilization and autonomous self-fertilization (CROSS treatment). The SELF_AUTO_ treatment was repeated on five flowers per plant, while the SELF_GEITO_ and CROSS treatments were each repeated on ten flowers per plant. Fruits were collected when ripe. Since SELF_GEITO_ treatments were performed before CROSS treatments, selfed seeds matured before crossed seeds, but the time lag did not affect seed-set (see Methods and Results S2). Each experimental flower was checked for presence or absence of a seed (binary trait: each flower can produce only one seed). The pollen type of the pollen-donor plants in the CROSS treatment of the pollen-dimorphic population I-1 was not recorded. Fortunately, this omission should not affect the interpretation of results, because the two pollen types of I-1 did not cause incompatibility reactions of different strengths, as demonstrated below (see also Method and Results S2). If A and B pollen triggered incompatibility reactions of different strengths, we would expect the variance of seed-set after outcrossing treatment in I-1 to be larger than the variance of seed-set after outcrossing treatment across all pollen-monomorphic populations I-4 (10 plants, all B pollen), I-3 (8 plants, all B pollen), M-1 (9 plants, all B pollen), M-2 (10 plants, all B pollen) and M-4 (10 plants, all A pollen); see Results and Fig. 5e, Supplementary Fig. S3). Contrary to this expectation (hence, non-significant: N = 5, *Z* = 2.032, *P* = 0.979), we found that outcrossed seed-set was less variable in the pollen-dimorphic (σ^2^ = 0.005) than pollen-monomorphic populations (M ± SE: 0.033 ± 0.014; details in Results S2). Consequently, pollen type (whether A or B) did not appreciably affect seed-set after outcrossing treatment in the dimorphic population I-1.

Previous studies concluded that specific combinations of pollen and stigma traits predict whether a species is SI or SC in *Limonieae* (Fig. 1)^6,14,32,33,37^, with SI leakiness recorded only in *Armeria maritima*^43^. Therefore, it was hypothesized that crosses between A pollen and pap stigmas and between B pollen and cob stigmas, expected to be compatible, should produce high seed-set (for definition, see above), while crosses between A pollen and cob stigmas and between B pollen and pap stigmas, expected to be incompatible, should produce very low seed-set (Fig. 1). To test these predictions and determine whether newly discovered plant types were SI or SC (see “Results” and Figs. 4 and 5), we compared seed-set of selfed and cross-pollinated flowers using a GLMM (using binomial error distribution and logit link function) with contrasts and *pollination treatment* (SELF_AUTO_, SELF_GEITO_, and CROSS), *plant type* (previously undescribed and typical), and their interaction as fixed effects. The reproductive output of pap and cob flowers was similar when pollinated with compatible pollen^52,101^, allowing us to pool typical flower types in the analyses. In all GLMMs, we accounted for both (a) hierarchical data structure by using *plant* nested within *population* as random effects and (b) unbalanced data set by using Satterthwaite’s method to determine the approximate denominator degree of freedom. The performance of the models was assessed by comparing the observed vs. predicted classification and by plotting the Pearson residual vs. predicted probability for the binary trait presence/absence of a seed. All statistical analyses were performed in SPSS version 27.0.0 (IBM Corp., Armonk, NY, USA) and sequential Bonferroni corrections were used to adjust significance levels for multiple tests, if applicable.

### Test of Baker’s law and contribution of newly discovered plant-types to uniparental reproduction on islands

We discovered plants with novel combinations of pollen-stigma traits in *L. lobatum* and demonstrated that this species can produce seeds uniparentally (see “Results”). By leveraging the results of the analyses above, we tested the following hypotheses: (i) If Baker’s law applies to *L. lobatum*, island plants should have higher capacity for uniparental reproduction than mainland plants; (ii) more plants with newly discovered pollen-stigma combinations should occur on islands than mainland, and (iii) island plants with newly discovered pollen-stigma combinations should have higher capacity for uniparental reproduction than island plants with previously described reproductive morphologies.

(i) *Do island plants have higher capacity for uniparental reproduction than mainland plants? (test of Baker’s law)* - We predicted that seed-set from manual self-pollination treatments is higher on islands than mainland. Utilizing the data generated above, we tested this prediction by comparing seed-set of selfed and cross-pollinated flowers between islands and mainland using a GLMM (binomial error distribution and logit link function) with contrasts and *pollination treatment* (SELF_AUTO_, SELF_GEITO_, and CROSS), *population locality* (mainland and island), and their interaction as fixed effects.
(ii) *Do more plants with newly discovered pollen-stigma combinations occur on islands than the mainland? - *If plants of *L. lobatum* with newly described pollen-stigma combinations were more SC, hence more able to produce seeds uniparentally, and if island plants had higher capacity for uniparental reproduction than mainland plants (as demonstrated by our “Results”; see above), then islands might harbor more newly described plant types than the mainland. Therefore, we tested whether plants with newly discovered pollen-stigma combinations were overrepresented on the Canary Islands vs. the geographically closer Iberian mainland and the geographically distant Dead Sea shore, using a one-tailed, two-sample *t*-test and a one-tailed, one-sample *t*-test, respectively.
(iii) *Do island plants with newly discovered pollen-stigma combinations have higher capacity for uniparental reproduction than island plants with previously described pollen-stigma combinations? - *Finally, we predicted that island plants with novel pollen-stigma combinations contributed significantly more to uniparental seed production on islands than plants with previously described pollen-stigma combinations. To test this prediction, we tested whether island plants with newly described pollen-stigma combinations produced significantly more seeds when manually selfed (SELF_GEITO_) than island plants with previously described pollen-stigma combinations, using a GLMM (binomial error distribution and logit link function) with *plant type* (previously undescribed and typical) as fixed effect.

### Supplementary Information

Supplementary Information 1.

Supplementary Information 2.

## Data Availability

In Supplementary Information, raw data are available for quantified pollen and stigma traits (Supplementary Table S3) and pollination experiment—selfed vs. outcrossed seed-sets (Supplementary Table S4).
