# Supplementary material for: Island plants with newly discovered reproductive traits have higher capacity for uniparental reproduction, supporting Baker’s law
Source: Sci Rep. 2024 May 18;14:11392. doi: 10.1038/s41598-024-62065-4 (PMC11102434; doi:10.1038/s41598-024-62065-4)
Supplement: Supplementary file 1 — Supplementary Information 1. [file 41598_2024_62065_MOESM1_ESM.pdf]

SUPPLEMENTARY INFORMATION

Island plants with newly discovered reproductive traits have higher capacity for uniparental reproduction, supporting Baker's law

Barbara Keller, Barbara Alther, Ares Jiménez, Konstantina Koutroumpa, Emiliano Mora-Carrera, and Elena Conti

(A) METHODS & RESULTS

Methods & Results S1: Effect of floral age and position on variability of stigma traits within plants.

Methods & Results S2: Effect of timing of manual pollination and pollen type (one vs. two) on outcrossed seed-set.

(B) FIGURES

Extended Figure 3. Diversity of stigmatic papillae types discovered in *Limonium lobatum*.

Figure S1 Flower visitors of *Limonium lobatum*.

Figure S2 Flower and Inflorescence structure in *Limonium lobatum*.

Figure S3 Sampling and number of plants and flowers used to study pollen and stigma traits and seed set.

Figure S4 Pollen-stigma interactions within flowers.

Figure S5 Performance assessment of GLMMs.

Figure S6 Temporal variation of stigma traits.

(C) TABLES

Table S1 Details to the Canarian and Iberian populations.

Table S2 Results of GLMMs.

Table S3 Raw data of pollen and stigma traits (TYPICAL: pollen-stigma combinations previously described in *Limonieae*; PREV. UNDESCRIBED: pollen-stigma combinations that are new in *Limonieae*) (see separate file).

Table S4 Raw data of pollination experiment - selfed vs. outcrossed seed-sets (see separate file).

(A) METHODS & RESULTS

Methods & Results S1: Effect of developmental stage (i.e., age) and position on variation of stigma traits within plants

We observed intra-plant diversity of stigma traits (Figs 3-5). It is well known that developmental stage and position within inflorescences can affect the expression of floral traits [1,2]. In *Limonium*, corolla, stigmas, and anthers wither within less than 24 hours after flower opening. For morphological analyses, we collected freshly opened flowers at the same day, if possible (see Table S3a). Therefore, all experimental flowers were at the same developmental stage when analyzed. Within spikes and spikelets, flowers bloom from the proximal to the distal part. Consequently, the later blooming flowers occur more distal within a spike or spikelet. If positional effects affected intra-plant variability of stigma traits, we would expect stigma traits to differ systematically in early *vs.* late blooming flowers. To test this prediction, we randomly sowed a second batch of seeds from each of the five island populations in July 2012, repotted six seedlings per population, and raised a total of 30 plants as described in the main section of the paper. One plant died. We thus harvested a total of 264 flowers from 29 individuals over a period of 13 to 84 days (details in Table S3b). Ten flowers had less than three intact stigmas and were therefore excluded from the analysis. From the remaining 254 flowers, we determined stigma traits as described in the main section of the paper. Five out of 29 plants had homogeneous stigma traits (cob = 3 plants; pap = 2 plants), while 24 plants had variable stigma traits. In the latter group of plants, stigma traits did not produce consistent patterns of trait variation between early and late blooming flowers (Fig. S6), suggesting that positional effects should not have appreciably altered the results.

Methods & Results S2: Effects of timing of manual pollination and pollen type (one vs. two) on outcrossed seed-set

Effect of timing – Plants from populations I-1, I-4, I-5, M-1, M-2, and M-4 produced considerably more flowers than plants from populations I-2, I-3, M-3, and M-5. Therefore, the cross treatment (CROSS) could only be carried out on plants from the former set of populations (Fig. S3b). In addition, plants were cross-pollinated up to 19 days after they were manually selfed (islands: median = 16.5 days; mainland: median = 11.0 days; Table S4), which could affect the success of selfed vs. outcrossed seed formation [3,4]. If this time lag negatively affected outcrossed seed-set, we would expect a negative and significant correlation between seed-set of cross-pollinated flowers and the time lag between the two manual pollination treatments (number of days between $SELF_{GEITO}$ and CROSS) in BOTH localities. To test this prediction, we calculated Spearmans rho between *outcrossed seed-set* and *time lag* for mainland and island plants. The correlation between *seed set* and *time lag* was not significant neither for island (Spearmans rho = -0.035, N = 28, P = 0.860) nor mainland plants (Spearmans rho = 0.017, N= 29, P = 0.930) indicating that the time lag between $SELF_{GEITO}$ and CROSS should not have appreciably altered the results.

Effect of pollen type – If pollen and stigma trait combinations predicted SI, stigmas should be more compatible with one of the two dimorphic pollen types. In the manual pollination experiment, ten out of 57 cross-pollinated plants occurred in the pollen-dimorphic population I-1, while 47 plants occurred in pollen-monomorphic populations (I-4: 10 plants; I-5: 8 plants; M-1: 9 plants; M-2: 10 plants; and M-4: 10 plants; Figs 5d, S3b). Since we did not record the pollen type of the pollen donors in the CROSS treatment, we might have pollinated flowers with either A pollen, or B pollen, or both in the pollen-dimorphic population I-1. If the two pollen types triggered incompatibility reactions of different strengths, we would expect outcrossed seed-set to vary more in the pollen-dimorphic I-1 than the five pollen-monomorphic populations (I-4, I-3, M-1, M-2, and M-4). To test this prediction, we estimated for each population the variance in outcrossed seeds-set and tested

with a one-tailed one-sample Wilcoxon Signed Rank Test, whether outcrossed seed-set varied significantly more in pollen-dimorphic than pollen-monomorphic populations. The variance of seed-set after outcrossing treatment in the pollen-dimorphic population I-1was $\sigma^2 = 0.005$, while it was 0.033 ± 0.014 (M ± SE) in the pollen-monomorphic populations. Hence, the variance of seed-set after outcrossing treatment in the pollen-dimorphic population I-1 was significantly smaller than the variance of seed-set after outcrossing treatment across all pollen-monomorphic populations (N = 5, $Z = 2.032$, $P = 0.021$). However, since this result is in the opposite direction of our expectation (i.e., we expected the variance of seed-set after outcrossing treatment in I-1 to be larger than the variance of seed-set after outcrossing treatment across all pollen-monomorphic populations) the result is non-significant (P = 0.979). This finding indicates that the presence of two vs. one pollen type in the populations should not have appreciably altered our results.

References

1 Diggle, P. K. Modularity and intra-floral integration in metameric organisms: plants are more than the sum of their parts. *Philosophical Transactions of the Royal Society B: Biological Sciences* **369**, doi:10.1098/rstb.2013.0253 (2014).

2 Harder, L. D., Strelin, M. M., Clocher, I. C., Kulbaba, M. W. & Aizen, M. A. The dynamic mosaic phenotypes of flowering plants. *New Phytol.* **224**, 1021-1034, doi:10.1111/nph.15916 (2019).

3 Lloyd, D. G. Self- and Cross-Fertilization in Plants. II. The Selection of Self- Fertilization. *Int. J. Plant Sci.* **153**, 370-380, doi:10.2307/2995677 (1992).

4 Kalisz, S., Vogler, D. W. & Hanley, K. M. Context-dependent autonomous self-fertilization yields reproductive assurance and mixed mating. *Nature* **430**, 884-887 (2004).

5 Brullo, S. & Erben, M. The genus *Limonium* (Plumbaginaceae) in Greece. *Phytotaxa* **240**, 1-+ (2016).

6 Baker, H. G. Dimorphism and monomorphism in the Plumbaginaceae: II. pollen and stigmata in the genus *Limonium*. *Ann Bot* **17**, 433-445, doi:10.2307/42907205 (1953).

7 Erben, M. Karyotype differentiation and its consequences in Mediterranean *Limonium*. *Webbia* **4**, 409-417 (1979).

(B) FIGURES

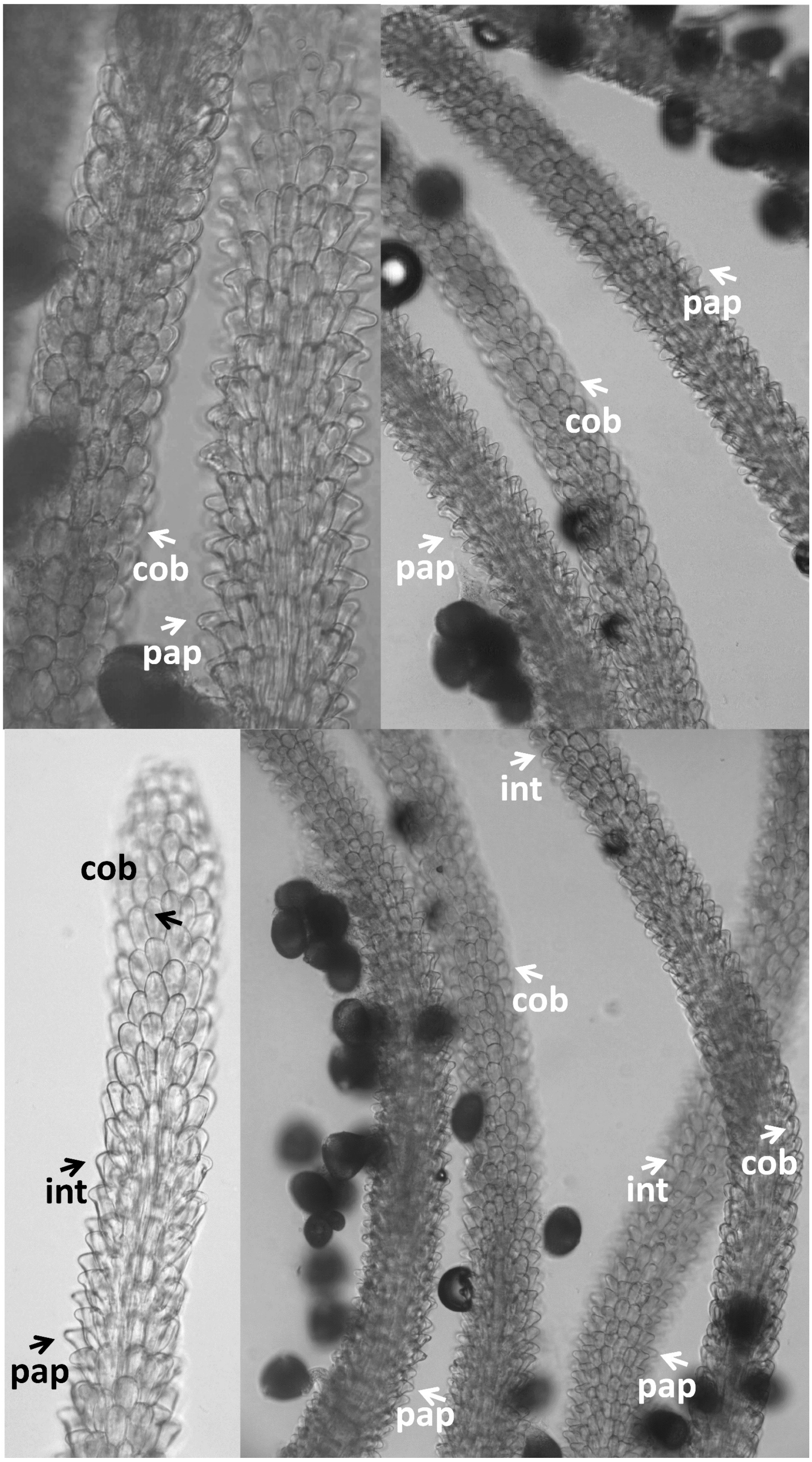

Extended Figure 3. Diversity of stigmatic papillae types discovered in *Limonium lobatum*. Stigmas with various combinations (*var* stigmas) of cob (flat shape), pap (protruding shape), and *int* (intermediate shape) papillae in the same stigma and among stigma of the same gynoecium. Arrows illustrate shapes of cob, pap and int papillae.

(a)

(b)

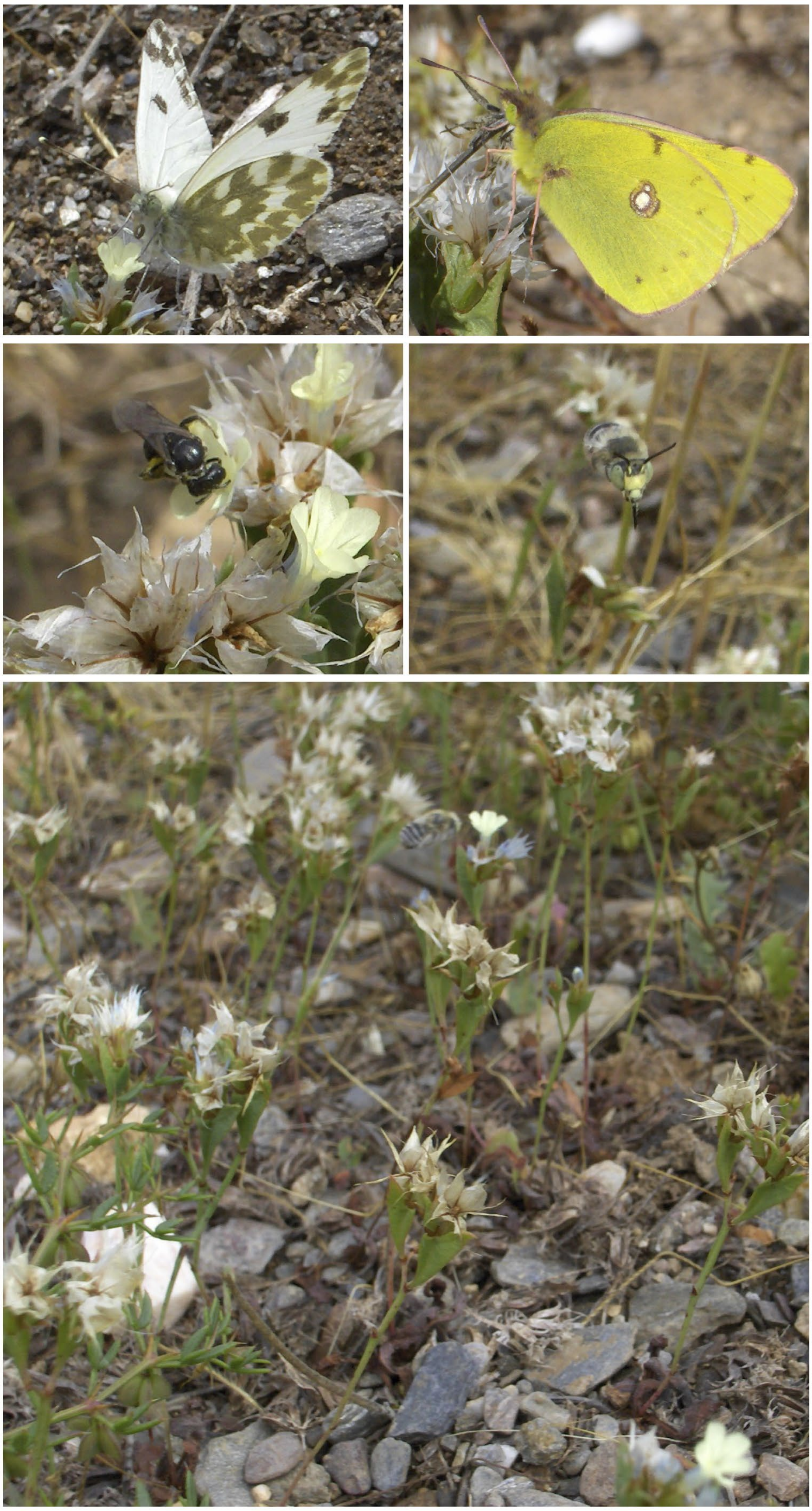

Figure S1. Flower visitors of *Limonium lobatum* in the Canary Islands and/or Iberian mainland. (a) Butterflies: *Pontia daplidice* (Linnaeus, 1758; left) and *Colias croceus* (Geoffroy in Fourcroy, 1785; right); **(b) Bees:** *Lassioglossum* sp. (left) and *Amegilla quadrifasciata* (right and bottom). Photo curtesy Ares Jiménez.

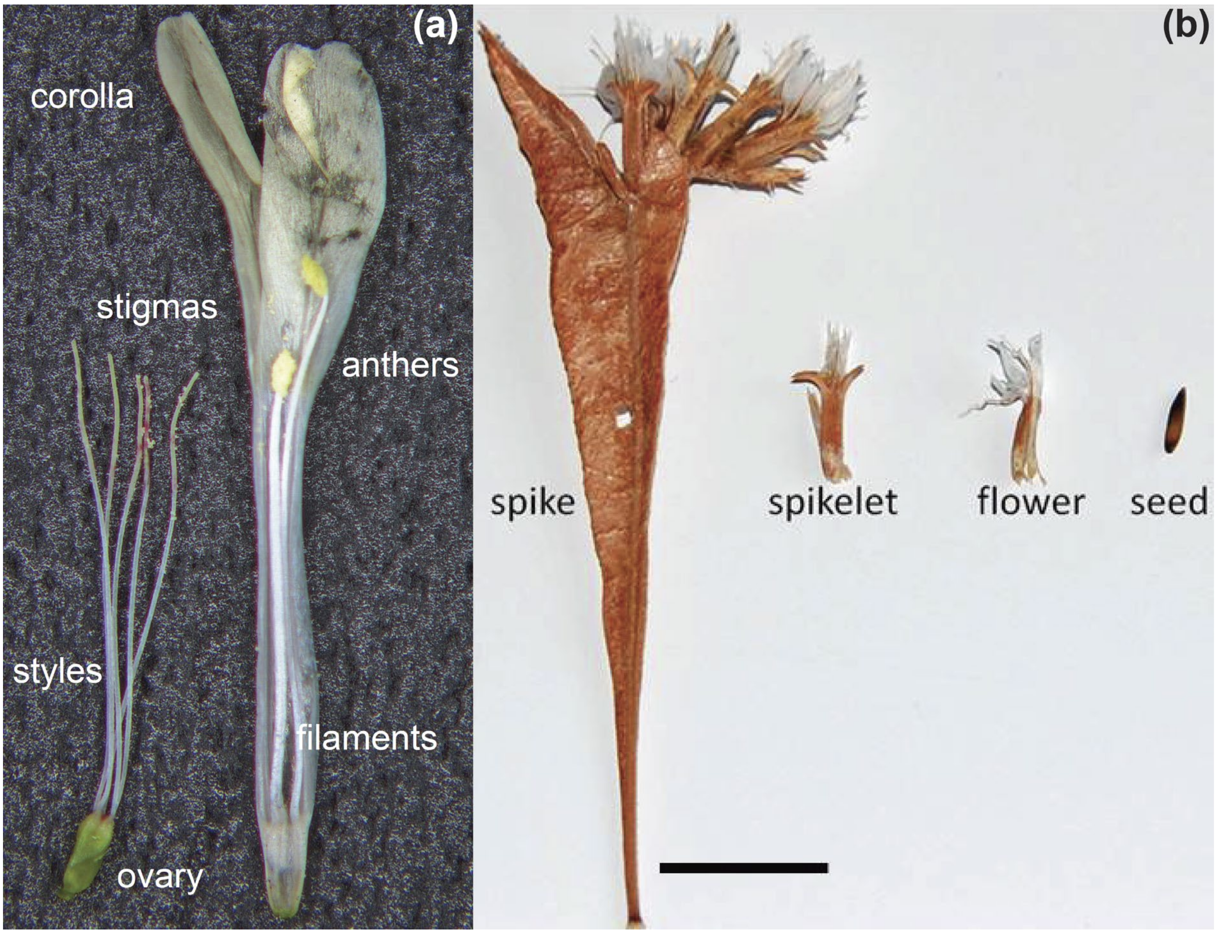

Figure S2. Flower (a) and inflorescence (b) structures in *Limonium lobatum*. (a) Left: dissected out gynoecium consisting of the ovary with one ovule, the five styles, and the five filiform (linear) stigmas; Right: dissected out corolla and androecium depicting two out of the five filaments and anthers. **(b)** From left to right: a spike comprises 6-12 spikelets; a spikelet comprises two to three flowers; a flower comprises a single ovule and produces one seed. Scale bar = 1 cm (see also species description by Brullo & Erben [5]).

	I-1	I-2	I-3	I-4	I-5	M-1	M-2	M-3	M-4	M-5	DS
Number of wild plants (seed parents)	20PL	20PL	20PL	20PL	20PL	20PL	20PL	20PL	20PL	20PL	–
Number of experimental plants raised from seedlings	20PL	20PL	20PL	20PL	20PL	20PL	20PL	20PL	20PL	20PL	10PL
**(a) Pollen and stigma traits**											
Pollen type: Pollen traits of 567 flowers from 167 plants	14PL 43FL	15PL 65FL	15PL 49FL	10PL 26FL	14PL 32FL	16PL 51FL	19PL 63FL	15PL 40FL	20PL 82FL	20PL 93FL	9PL 23FL
Gynoecium type: Stigma traits of 950 flowers from 203 plants [4723 individual stigmas]	17PL 81FL	20PL 97FL	20PL 95FL	20PL 96FL	20PL 98FL	20PL 97FL	19PL 90FL	19PL 87FL	20PL 92FL	20PL 95FL	8PL 22FL
Flower type: Pollen & stigma traits of 768 flowers from 165 plants	13PL 62FL	15PL 74FL	15PL 72FL	10PL 47FL	14PL 69FL	16PL 77FL	19PL 90FL	15PL 68FL	20PL 92FL	20PL 95FL	8PL 22FL
Plant type: Pollen & stigma traits of 768 flowers from 165 plants	13PL 62FL	15PL 74FL	15PL 72FL	10PL 47FL	14PL 69FL	16PL 77FL	19PL 90FL	15PL 68FL	20PL 92FL	20PL 95FL	8PL 22FL
**(b) Seed set**											
Autonomous selfing (AUTO): 810 flowers of 173 plants [max. 5 flowers per plant]	20PL 95FL	20PL 89FL	20PL 93FL	20PL 99FL	19PL 89FL	19PL 85FL	19PL 100FL	16PL 65FL	20PL 95FL	–	–
Geitonogamous selfing (GEITO): 1574 flowers of 164 plants [max. 10 flowers per plant]	20PL 198FL	20PL 191FL	15PL 135FL	20PL 199FL	19PL 184FL	19PL 186FL	18PL 170FL	13PL 116FL	20PL 195FL	–	–
Cross pollination (CROSS): 497 flowers of 57 plants [max. 10 flowers per plant]	10PL 92FL	–	–	10PL 92FL	8PL 77FL	9PL7 79FL	10PL 81FL	–	10PL 76FL	–	–

Figure S3. Sampling and number of plants and flowers used to characterize **(a)** pollen and stigma traits and **(b)** seed set of *Limonium lobatum* in five populations from the Canary Islands (I-1 to I-5, red quadrilaterals), five populations from the Iberian mainland (M-1 to M-5; green quadrilaterals), and one population from the Dead Sea (DS; blue quadrilaterals). Abbreviations: PL, plant; FL, flower; –, no data available; and abbreviations of populations as reported in legend of Fig. 2.

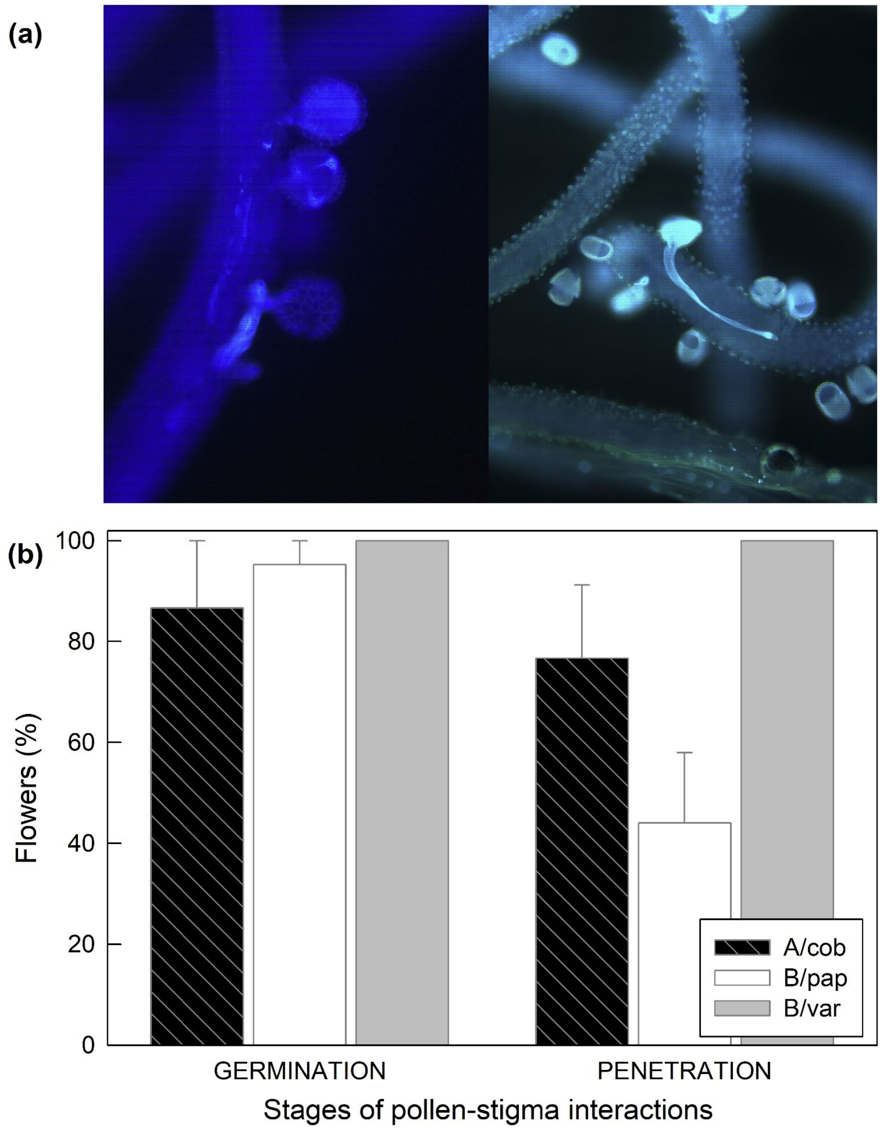

Figure S4. Pollen-stigma interactions within stigmas of *Limonium lobatum*. Previous studies demonstrated that an incompatibility system prevents the germination of pollen grains or, if pollen grains germinate, the penetration of the stigmatic surfaces by the pollen tubes in plants with cob stigmas and A pollen (A/cob) and plants with pap stigmas and B pollen (B/pap; Fig. 1) [6,7]. **(a)** Pictures of A pollen on cob stigmas (left panel) and B pollen on pap stigmas (right panel). **(b)** Stages of pollen-stigma interactions: mean values and standard errors of relative number of self-pollinated flowers per plant with germinated pollen grains (GERMINATION) and pollen tubes that have penetrated the stigmatic surfaces (PENETRATION). For definitions of pollen and stigma traits, see Fig. 4.

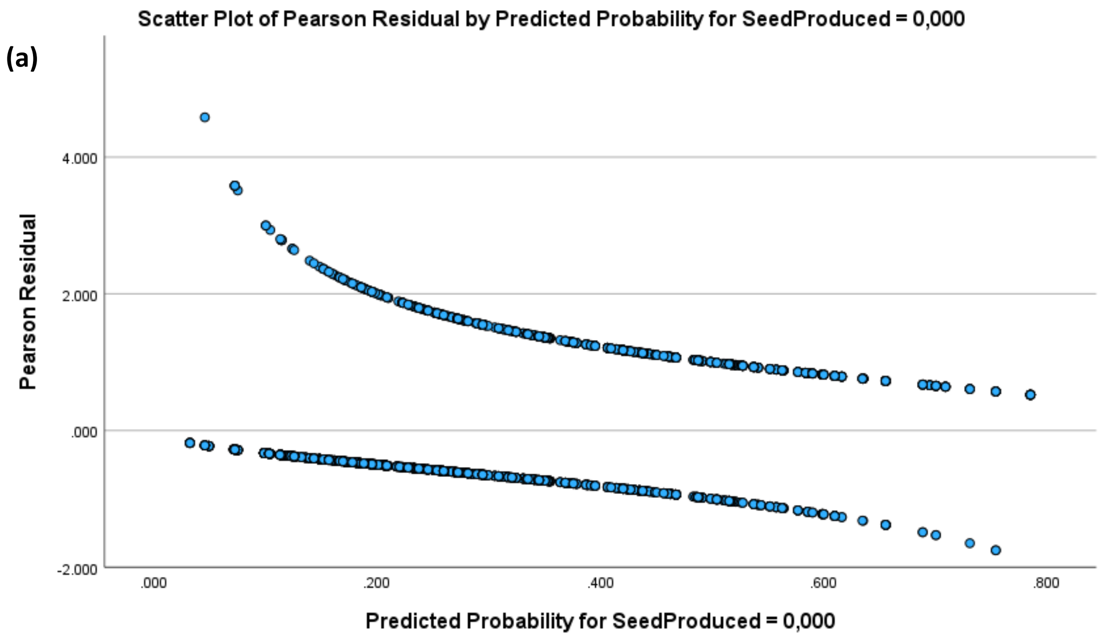
Scatter Plot of Pearson Residual by Predicted Probability for SeedProduced = 0,000
(a)
Pearson Residual
4.000
2.000
.000
-2.000
.000
.200
.400
.600
.800
Predicted Probability for SeedProduced = 0,000

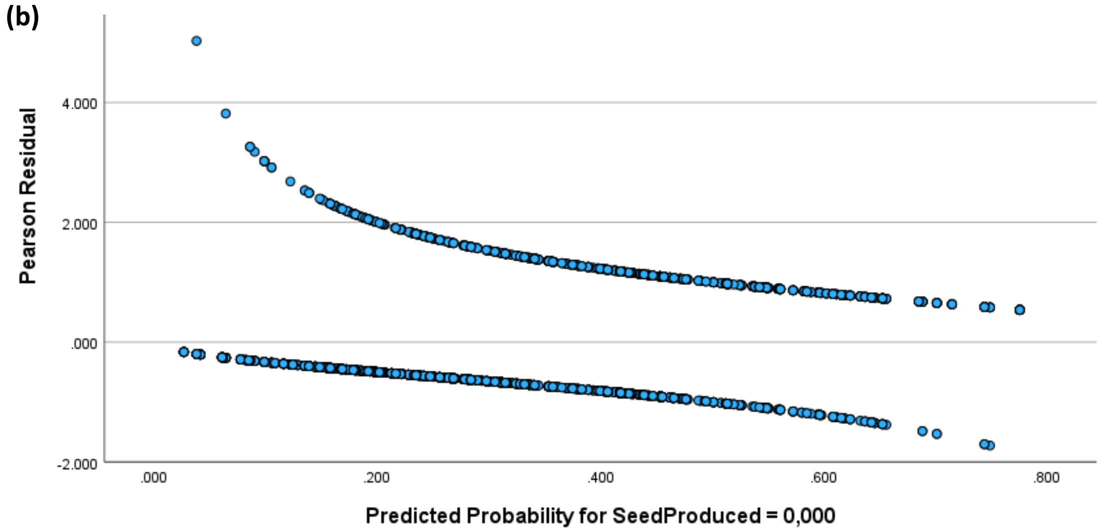
(b)
Pearson Residual
4.000
2.000
.000
-2.000
.000
.200
.400
.600
.800
Predicted Probability for SeedProduced = 0,000

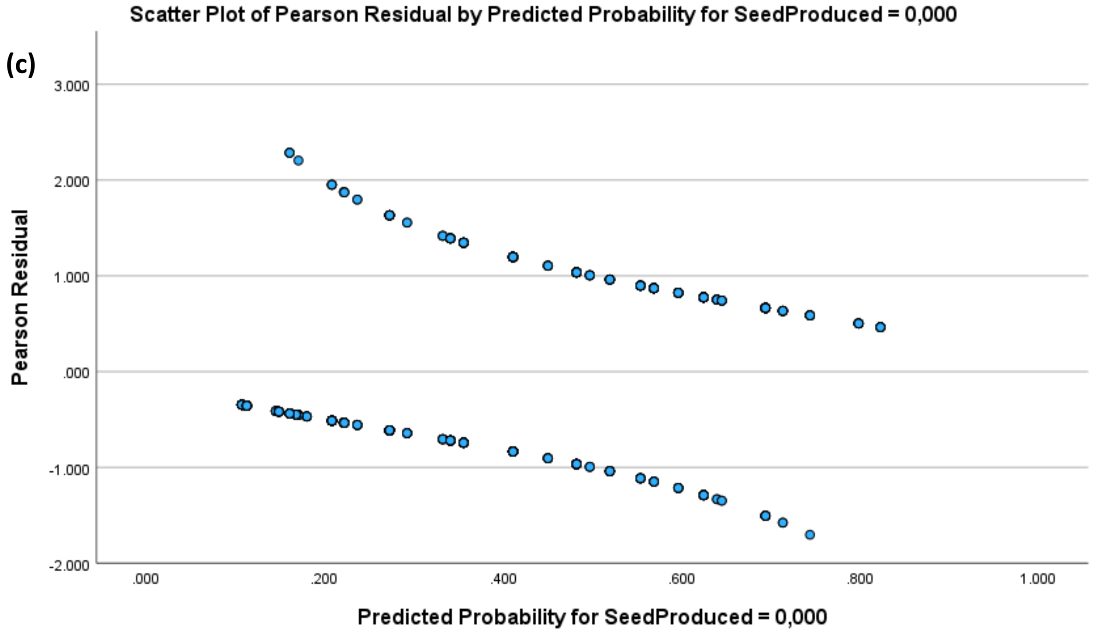
Scatter Plot of Pearson Residual by Predicted Probability for SeedProduced = 0,000
(c)
Pearson Residual
3.000
2.000
1.000
.000
-1.000
-2.000
.000
.200
.400
.600
.800
1.000
Predicted Probability for SeedProduced = 0,000

Figure S5 (previous page). Plots depicting Pearson Residual vs. Predicted Probability of Seed production, as estimated from generalized linear mixed effects models (GLMM), testing whether seed-set differed **(a)** among *pollination treatments* (autonomous selfing, geitonogamous selfing, and cross-pollination) and *plant types* (PREV. UNDESCRIBED vs. TYPICAL, subdivided into pollen-stigma combinations previously described as self-incompatible (A/cob & B/pap) and self-compatible (B/cob & A/pap), **(b)** among *pollination treatments* (autonomous selfing, geitonogamous selfing, and cross-pollination) and *location* (island and mainland), and **(c)** between manually selfed plant types (previously undescribed & previously described) on the islands.

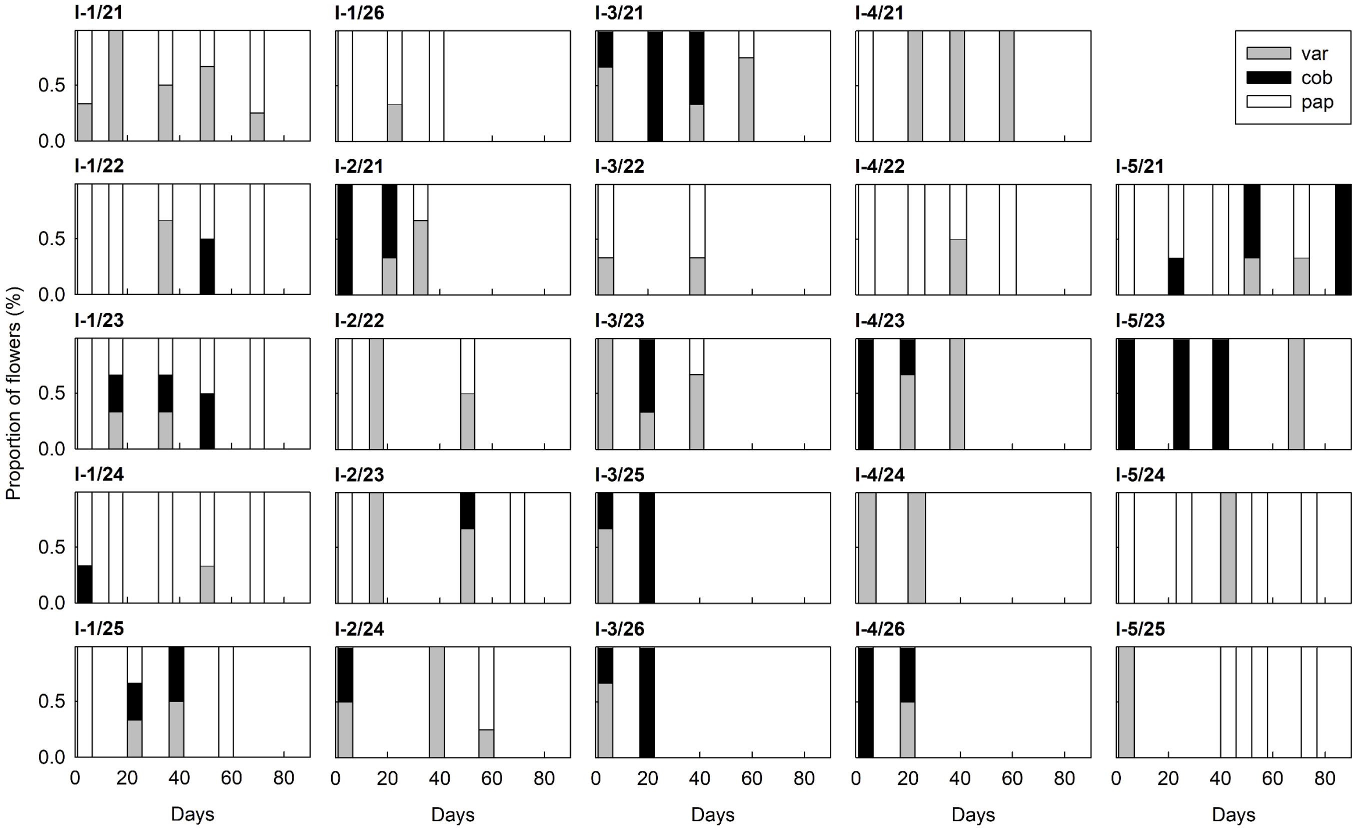

Figure S6. Temporal variation of stigma traits of individual island plants of *Limonium lobatum*. Abbreviations: pap = all stigmas of a flower with uniform protruding stigmatic papillae cells; cob = all stigmas of a flower with uniformly flat stigmatic papillae cells, var = either all stigmas of a flower with variable shapes of papillae cells or at least one stigma different from the others (see also Fig. 4). Abbreviations of populations as reported in legend of Fig. 2.

(C) TABLES

Table S1. Populations of *Limonium lobatum* from the Canary Islands (I) and the Iberian mainland (M) analysed in this study. Voucher specimens were deposited at the herbarium of Z, Zürich, Switzerland.

Population abbreviation	Location	Coordinates	Altitude (m.a.s.l.)	Collectors	Collector number	Herbarium voucher
I-1	Antigua	28° 22' 39.4" N<br>13° 58' 46.2" W	171	A. Jiménez,<br>A. Santos-Guerra	AJ 258	Z-000227005
I-2	Betancuria	28° 24' 04.1" N<br>14° 03' 45.4" W	320	A. Jiménez,<br>A. Santos-Guerra	AJ 259	Z-000227004
I-3	Tarajalejo	28° 14' 12.3" N<br>14° 05' 14.9" W	71	A. Jiménez,<br>A. Santos-Guerra	AJ 260	Z-000227006
I-4	Esquinzo	28° 04' 47.2" N<br>14° 17' 56.3" W	30	A. Jiménez,<br>A. Santos-Guerra	AJ 261	Z-000227018
I-5	El Viso	28° 10' 13.4" N<br>16° 28' 59.8" W	425	A. Jiménez	AJ 272	Z-000227016
M-1	Castillo de Macenas	37º 04' 45.0" N<br>01º 51' 03.8" W	8	A. Jiménez	AJ 218	Z-000227012
M-2	Tabernas	37º 1' 17.6" N<br>02º 25' 51.5" W	323	A. Jiménez	AJ 221	Z-000227013
M-3	Cala Blanca	37º 29' 01.3" N<br>01º 27' 47.7" W	4	A. Jiménez	AJ 211	Z-000227011
M-4	Los Cocedores	37º 22' 34.7" N<br>01º 37' 43.3" W	9	A. Jiménez	AJ 215	Z-000227002
M-5	Los Nietos	37º 39' 28.2" N<br>0º 48' 08.8" W	9	A. Jiménez	AJ 209	Z-000227015

Table S2. Results of generalized linear mixed effects models (GLMMs) and contrasts, testing whether seed-set differed **(a)** among *pollination treatments* (autonomous selfing, geitonogamous selfing, and cross-pollination) and *plant types* (PREV. UNDESCRIBED vs. TYPICAL, subdivided into pollen-stigma combinations previously described as self-incompatible (A/cob & B/pap) and self-compatible (B/cob & A/pap), **(b)** among *pollination treatments* (autonomous selfing, geitonogamous selfing, and cross-pollination) and *location* (island and mainland), and **(c)** between *plant types* (PREV. UNDESCRIBED vs. TYPICAL) on the islands. **(a)** on 2608 flowers of 156 plants (69 PREV. UNDESCRIBED and 87 TYPICAL), **(b)** based on 2881 flowers of 174 plants (69 PREV. UNDESCRIBED, 87 TYPICAL, and 18 undetermined), and **(c)** based on 802 flowers of 83 plants (61 PREV. UNDESCRIBED and 22 TYPICAL).

	Source	*F*	*d.f.*	*P*	Contrasts	(test statistics, *t*; *d.f.*; adjusted p-values)	
**(a)**	Plant type (P)	0.295	2, 178	0.745			
	Treatment (T)	16.292	2, 2599	<0.001			
	P × T	6.538	4, 2599	<0.001	$SELF_{AUTO}$	$SELF_{GEITO}$	CROSS
	TYPICAL, prev. described as SI *vs*. SC				-0.214; 423; 1.000	0.328; 211; 0.743	0.659; 1030; 0.510
	PREV. UNDESCR. *vs*. TYPICAL, prev. described as SI				0.788; 421; 1.000	2.139; 195; 0.067	-2.469; 1269; 0.040
	PREV. UNDESCR. *vs*. TYPICAL, prev. described as SC				0.589; 394; 1.000	2.593; 201; 0.031	-2.480; 786; 0.040
**(b)**	Location (L)	1.889	1, 208	0.171			
	Treatment (T)	25.982	2, 2875	<0.001			
	L × T	22.822	2, 2875	<0.001	$SELF_{AUTO}$	$SELF_{GEITO}$	CROSS
	island vs. mainland				1.827; 462; 0.068	2.601; 222 0.010	-4.705; 905; <0.001
**(c)**	Plant type (P)	6.276	1, 76	0.014			

SI, self-incompatible, SC, self-compatible, $SELF_{AUTO}$, autonomous selfing; $SELF_{GEITO}$, geitonogamous selfing; CROSS, cross pollination. The models predicted the binary trait seed present/absent correctly in 73.6%, 73.8%, and 73.1% of all flowers in **(a), (b),** and **(c)**, respectively.

Table S3 Raw data of pollen and stigma traits (TYPICAL: pollen-stigma combinations previously described in *Limonioideae*; PREV. UNDESCRIBED: pollen-stigma combinations that are new in *Limonioideae*) (see separate file).

Table S4 Raw data of pollination experiment - selfed vs. outcrossed seed-sets (see separate file).
